# Molecular docking and MD simulation studies of 4-thiazol-*N*-(pyridin-2-yl)pyrimidin-2-amine derivatives as novel inhibitors targeted to CDK2/4/6

**DOI:** 10.1007/s00432-024-05818-y

**Published:** 2024-06-10

**Authors:** Jia-Dong Liang, Yu-E Zhang, Fei Qin, Wan-Na Chen, Wen-Mei Jiang, Zeng Fang, Xiao-Li Liang, Quan Zhang, Jie Li

**Affiliations:** 1 Department of Head and Neck Surgery, State Key Laboratory of Oncology in South China, Collaborative Innovation Center for Cancer Medicine, Guangdong Provincial Clinical Research Center for Cancer, Sun Yat-Sen University Cancer Center, Guangzhou, 510060 People’s Republic of China; 2 Department of Breast and Thyroid Surgery, Guangzhou Women and Children’s Medical Center, 9 Jinsui Road, Guangzhou, 510623 Guangdong People’s Republic of China; 3 Department of Thyroid and Breast Surgery, The First Affiliated Hospital, Sun Yat-Sen University, Guangzhou, 510000 People’s Republic of China; 4 Department of Pharmacy, The Affiliated Jiangmen TCM Hospital of Jinan University, No. 30 Huayuan East Road, Jiangmen, 529000 People’s Republic of China; 5 Department of Nursing, The Linyi Mental Health Center, Linyi, People’s Republic of China

**Keywords:** Cyclin-dependent kinase_1_, Breast carcinoma_2_, Molecular dynamics simulation_3_, Molecular docking_4_, Inhibitors_5_

## Abstract

**Purpose:**

Nowadays, cyclin-dependent kinase 4/6 (CDK4/6) inhibitors have been approved for treating metastatic breast cancer and have achieved inspiring curative effects. But some discoveries have indicated that CDK 4/6 are not the requisite factors in some cell types because CDK2 partly compensates for the inhibition of CDK4/6. Thus, it is urgent to design CDK2/4/6 inhibitors for significantly enhancing their potency. This study aims to explore the mechanism of the binding of CDK2/4/6 kinases and their inhibitors to design novel CDK2/4/6 inhibitors for significantly enhancing their potency in different kinds of cancers.

**Materials and methods:**

A series of 72 disparately functionalized 4-substituted *N*-phenylpyrimidin-2-amine derivatives exhibiting potent inhibitor activities against CDK2, CDK4 and CDK6 were collected to apply to this research. The total set of these derivatives was divided into a training set (54 compounds) and a test set (18 compounds). The derivatives were constructed through the sketch molecule module in SYBYL 6.9 software. A Powell gradient algorithm and Tripos force field were used to calculate the minimal structural energy and the minimized structure was used as the initial conformation for molecular docking. By the means of 3D-QSAR models, partial least squares (PLS) analysis, molecular dynamics (MD) simulations and binding free energy calculations, we can find the relationship between structure and biological activity.

**Results:**

In this study, we used molecular docking, 3D-QSAR and molecular dynamics simulation methods to comprehensively analyze the interaction and structure–activity relationships of 72 new CDK2/4/6 inhibitors. We used detailed statistical data to reasonably verify the constructed 3D-QSAR models for three receptors (q^2^ of CDK2 = 0.714, R^2^_pred_ = 0.764, q^2^ = 0.815; R^2^_pred_ of CDK4 = 0.681, q^2^ = 0.757; R^2^_pred_ of CDK6 = 0.674). MD simulations and decomposition energy analysis validated the reasonability of the docking results and identified polar interactions as crucial factors that influence the different bioactivities of the studied inhibitors of CDK2/4/6 receptors, especially the electrostatic interactions of Lys33/35/43 and Asp145/158/163. The nonpolar interaction with Ile10/12/19 was also critical for the differing potencies of the CDK2/4/6 inhibitors. We concluded that the following probably enhanced the bioactivity against CDK2/4/6 kinases: (1) electronegative groups at the N1-position and electropositive and moderate-sized groups at ring E; (2) electrogroups featured at R_2_; (3) carbon atoms at the X-position or ring C replaced by a benzene ring; and (4) an electrogroup as R_4_.

**Conclusion:**

Previous studies, to our knowledge, only utilized a single approach of 3D-QSAR and did not integrate this method with other sophisticated techniques such as molecular dynamics simulations to discover new potential inhibitors of CDK2, CDK4, or CDK6. So we applied the intergenerational technology, such as 3D-QSAR technology, molecular docking simulation techniques, molecular dynamics simulations and MMPBSA19/MMGBSA20-binding free energy calculations to statistically explore the correlations between the structure with biological activities. The constructed 3D-QSAR models of the three receptors were reasonable and confirmed by the excellent statistical data. We hope the results obtained from this work will provide some useful references for the development of novel CDK2/4/6 inhibitors.

## Introduction

Cyclin-dependent kinases (CDKs) which can produce some extracellular effects, are classified as serine/threonine protein kinases. In mammalian cells, some well-differentiated CDKs can dominate the sequential nature and inactivation of cell cycle progression (Bury et al. 2021; Morrison et al. 2024), which is mediated by an intricate system of regulatory factors, including the tumor depressor retinoblastoma protein 1 (RB1) (Cornwell et al. 2023).

CDK4/6 are key regulators of the cell cycle, forming a complex with cyclin, phosphorylating the retinoblastoma gene (Rb), and releasing transcription factor E2F, which can trigger the cell cycle transition from the G1 phase to the S1 phase (Ammazzalorso et al. 2021). In hormone receptor (HR) positive and human epidermal growth factor receptor 2 (HER2)-triple-negative breast cancer, the overexpression of CDK4 and 6 can lead to uncontrolled cell proliferation that may evolve into malignant tumors (Zabihi et al. 2023). CDK4 and 6 inhibitors can block CDK4 and 6 kinase activity, which reduces the phosphorylation level of the Rb protein, inhibiting transcription factors to inhibit cancer cell division and proliferation, restore cell cycle control, block tumor cell proliferation, and inhibit breast cancer cell growth (Rubin et al. 2020).

CDK4/6 inhibitors induce tumor cell cycle arrest and synergistically promote antitumor immune responses. CDK4/6 inhibitors enhance the expression of genes that control the processing and presentation of antigens on the surface of cancer cells, inhibit the Rb-EF2 pathway (Goel et al. 2022), reduce the activity of DNA methyltransferases, inhibit the proliferation of regulatory T cells, prime cytotoxic T lymphocytes for eliminating tumor cells and heighten their antitumor functions (Morrison et al. 2024).

In light of the success of cyclin-dependent kinase 4 and 6 inhibitors in advanced HR-positive and HER2-triple-negative breast cancer, researchers have been striving to find more uses under different circumstances, including for early stage HR-positive and HER2-triple-negative breast cancer (Chou et al. 2020; Haddad et al. 2023) and for clinical breast oncology in other subtypes, such as HER2-positive breast cancer, HER2-positive estrogen receptor (ER)-positive breast cancer and triple-negative breast cancer (TNBC) (Blohmer et al. 2022; Tolaney et al. 2020).

In addition to the cyclin-dependent kinases 4 and 6, CDK2 is an extensively studied target for eliminating carcinomas because of its vital functions in signal transduction in cell-cycle control (Tadesse et al. 2020; Freeman-Cook et al. 2021). For some reasons CDK4/6 does not have a key role in mediating the proliferation of different cell types, likely because CDK2 can partly compensate for the inhibition of CDK4/6 in diverse cells (Arora et al. 2023). In several in vitro models, it has been shown that cells resistant to palbociclib also exhibit resistance to ribociclib, while still demonstrating sensitivity to abemaciclib. The observed absence of resistance might perhaps be related to the simultaneous suppression of CDK2 (Morrison et al. 2024).

CDK2, CDK4 and CDK6 are folded similarly with high sequence identity, expressed diffusely in the human body (Al-Warhi et al. 2020). Additionally, despite their structural similarities and widespread expression throughout the human body, there are four key residue differences in the ATP-binding sites of CDK2 (Glu12, Lys33, Lys89 and Asp145), CDK4 (Val14, Lys35, Arg101 and Asp158) and CDK6 (Glu21, Lys43, Thr106 and Asp163). These residue differences in the binding pockets may offer opportunities for the development of selective inhibitors against CDK2, CDK4 and CDK6.

Molecular modeling, serving as an effective means of new drug synthesis, has been applied to CDK2, CDK4 and CDK6 inhibitors. Specifically, structure–activity relationships (SARs) of some known CDK2 inhibitors have been analyzed on 3D-QSAR model platforms by statistically utilizing a comparative molecular field analysis (CoMFA) method (Moussaoui et al. 2023; Abdel-Rahman et al. 2021). From another perspective, previous research of 3D-QSAR models has simply considered the advantages of the structural features, aiming only to determine the maximum activity of a specific target protein and neglecting differences among homologous proteins. Besides, other previous studies found that 4-thiazol-*N*-(pyridin-2-yl)pyrimidin-2-amine derivatives were reported to be promising inhibitors of these three cyclin-dependent kinases (Chohan et al. 2016; Zheng et al. 2014). However, previous studies, to our knowledge, only utilized a single approach of 3D-QSAR and did not integrate this method with other sophisticated techniques such as molecular dynamics simulations to discover new potential inhibitors of CDK2, CDK4, or CDK6.

Encouraged by the previous outcomes from other laboratories, we applied the intergenerational technology, such as 3D-QSAR technology, molecular docking simulation techniques, molecular dynamics simulations and MMPBSA19/MMGBSA20-binding free energy calculations to statistically determine relevant biological activities of dozens of compounds. These compounds contained diverse chemical properties, including steric and electrostatic fields.

In this study, we used the application technique described above to evaluate the interaction of the core structure of 4-substituted *N*-phenylpyrimidin-2-amines with CDK2, CDK4 and CDK6 for identifying potential lead compounds that can inhibit these three kinases. Herein, a combination of 3D-QSAR, docking and MD simulations methods was applied to verify the effectiveness of disparate interactional fields for use in fostering the development of highly potent selective CDK2/4/6 inhibitors.

## Materials and methods

### Data set

A series of 72 disparately functionalized 4-substituted *N*-phenylpyrimidin-2-amine derivatives exhibiting potent inhibitor activities against CDK2, CDK4 and CDK6 were collected to apply to this research (Tadesse et al. 2017a, 2018, 2017b) (Table 1).

**Table 1:** Molecular structure of compounds with their experimental and predictive activities

No.	R_1_	R_2_	R_3_	R4	X	PKi		
						CDK2	CDK4	CDK6
1	NH-Cyclopentyl	CH_3_	H	CH_2_NH	N	6.556	9.000	8.097
2	NH-Cyclopentyl	CH_3_	H	NCH_3_	N	6.686	8.699	8.046
3*	NH-Cyclopentyl	CH_3_	H	NCH_2_CH_3_	N	6.450	8.699	7.959
4	NH-Cyclopentyl	CH_3_	H	NCOCH_3_	N	6.110	8.222	7.032
5	NH-Cyclopentyl	CH_3_	H	O	N	5.329	8.398	7.523
6	NH-Cyclopentyl	CH_3_	H	CHNH_2_	N	6.742	8.523	6.876
7	NH-Cyclopentyl	CH_3_	H	NSO_2_CH_3_	N	7.114	8.155	7.260
8	NCH_3_-Cyclopentyl	CH_3_	H	NH	N	6.759	8.699	8.000
9*	NCH_3_-Cyclopentyl	CH_3_	H	NCH_3_	N	6.322	8.699	8.000
10*	NCH_3_-Cyclopentyl	CH_3_	H	NCOCH_3_	N	6.917	8.000	7.509
11	NCH_3_–C_6_H_5_	CH_3_	H	NCH_3_	N	5.983	7.585	7.000
12*	NH–CH(CH_3_)_2_	CH_3_	H	NH	N	6.620	8.301	7.959
13	NH–CH(CH_3_)_2_	CH_3_	H	CH_2_NH	N	6.097	7.796	7.553
14	NH–CH(CH_3_)_2_	CH_3_	H	NCH_3_	N	6.399	8.523	7.824
15*	NH–CH(CH_3_)_2_	CH_3_	H	NCOCH_3_	N	6.699	7.678	6.979
16*	NH–CH(CH_3_)_2_	CH_3_	H	O	N	6.896	7.387	7.086
17	NH-Cyclopentyl	CF_3_	H	NH	N	7.658	8.097	8.699
18	NH-Cyclopentyl	CF_3_	H	NCH_3_	N	7.658	9.000	8.523
19	NH-Cyclopentyl	CF_3_	H	NCOCH_3_	N	7.854	8.398	8.222
20	NH-Cyclopentyl	CF_3_	H	O	N	6.812	8.097	7.959
21*	NH-Cyclopentyl	CF_3_	H	NCHO	N	7.824	7.959	8.155
22	NH-Cyclopentyl	CH_3_	F	NH	N	6.592	8.523	8.155
23	NH-Cyclopentyl	CH_3_	F	CH_2_NH	N	6.380	7.854	8.000
24	NH-Cyclopentyl	CH_3_	F	NCH_3_	N	6.987	9.000	8.523
25*	NH-Cyclopentyl	CH_3_	F	NCH_2_CH_3_	N	6.457	8.699	8.222
26	NH-Cyclopentyl	CH_3_	F	NCOCH_3_	N	6.642	7.469	7.638
27*	NH-Cyclopentyl	CH_3_	F	O	N	6.983	8.222	7.699
28	NH-Cyclopentyl	CH_3_	F	NSO_2_CH_3_	N	6.458	7.409	6.996
29	NH-Cyclopentyl	CH_3_	F	CHN(CH_3_)_2_	N	5.939	9.000	7.509
30	NHCH_3_	CH_3_	CN	NBn	CH	7.770	9.000	7.444
31	NHCH_3_	CH_3_	H	NBn	CH	7.432	8.222	6.648
32*	NHCH_3_	CH_3_	H	NH	N	6.051	8.222	6.943
33	NHCH_3_	CH_3_	H	NCH_3_	N	6.187	8.301	7.301
34	NHCH_3_	CH_3_	H	NCOCH_3_	N	6.509	6.238	5.518
35*	NHCH_3_	CH_3_	H	O	N	6.338	7.699	7.215
36	NHCH_3_	CH_3_	CN	NH	N	6.674	9.000	7.398
37*	NHCH_3_	CH_3_	CN	NCH_3_	N	6.521	8.398	7.495
38*	NHCH_3_	CH_3_	CN	NCOCH_3_	N	5.350	8.398	7.523
39*	NHCH_3_	CH_3_	CN	O	N	6.697	8.398	7.194
40	NHCH_3_	CH_3_	F	NH	N	6.627	8.398	7.495
41	NHCH_3_	CH_3_	F	NCH_3_	N	6.438	8.699	8.000
42	NHCH_3_	CH_3_	F	NCH_2_CH_3_	N	6.177	8.301	7.699
43	NHCH_3_	CH_3_	F	NCOCH_3_	N	6.750	7.770	7.337
44	NHCH_3_	CH_3_	F	O	N	7.000	8.301	7.523
45	NHCH_3_	CH_3_	F	NSO_2_CH_3_	N	7.119	7.432	6.527
46	NHCH_3_	CH_3_	F	CHN(CH_3_)_2_	N	5.477	8.699	6.554
47	NHCH_3_	CH_3_	F	CH_2_NH_2_	N	6.686	8.523	7.495
48*	N(CH_3_)_2_	CH_3_	F	NH	N	6.475	8.398	7.398
49	N(CH_3_)_2_	CH_3_	F	NCH3	N	5.857	8.699	7.260
50*	N(CH_3_)_2_	CH_3_	F	NCOCH_3_	N	5.983	7.620	6.437
51	N(CH_3_)_2_	CH_3_	H	NH	N	6.044	7.678	7.252
52	N(CH_3_)_2_	CH_3_	H	NCH3	N	5.845	7.523	6.812
53	N(CH_3_)_2_	CH_3_	H	NCOCH3	N	6.011	7.060	6.631
54	CH_3_	CH_3_	H	NAc	N	5.712	7.523	6.770
55	CH_2_CH_3_	CH_3_	H	NH	N	5.767	7.229	6.625
56	CH_2_CH_3_	CH_3_	H	NCH_3_	N	5.740	7.620	6.009
57	CH_2_CH_3_	CH_3_	H	NCH_2_CH_3_	N	5.745	8.000	5.777
58	CH_2_CH_3_	CH_3_	H	O	N	5.631	6.745	5.764
59	CH(CH_3_)_2_	CH_3_	H	NH	N	6.618	7.959	7.523
60*	CH(CH_3_)_2_	CH_3_	H	NCH_3_	N	6.542	8.000	7.538
61	CH(CH_3_)_2_	CH_3_	H	NCH_2_CH_3_	N	6.333	8.301	7.602
62	CH(CH_3_)_2_	CH_3_	H	NAc	N	6.609	7.208	6.680
63*	CH(CH_3_)_2_	CH_3_	H	O	N	7.222	7.959	6.174
64	OCH_3_	CH_3_	H	NCH_3_	N	5.438	6.509	6.029
65	SCH_3_	CH_3_	H	NH	N	6.143	8.301	7.699
66	SCH_3_	CH_3_	H	NCH_3_	N	5.569	8.155	7.377
67	SCH_3_	CH_3_	H	NAc	N	5.322	6.721	5.708
68	SCH(CH_3_)_2_	CH_3_	H	NH	N	6.745	7.523	6.699
69	SCH(CH_3_)_2_	CH_3_	H	NCH_3_	N	6.745	9.000	7.824
70	SCH(CH_3_)_2_	CH_3_	H	NAc	N	7.125	8.301	7.699
71	NHCH_3_	CH_3_	H	NBn	N	5.301	6.772	5.567
72	NH-Cyclopentyl	CH_3_	H	CH_2_	N	5.301	7.155	6.590

The compounds with “*” are included in the test set

**Fig. 1:**
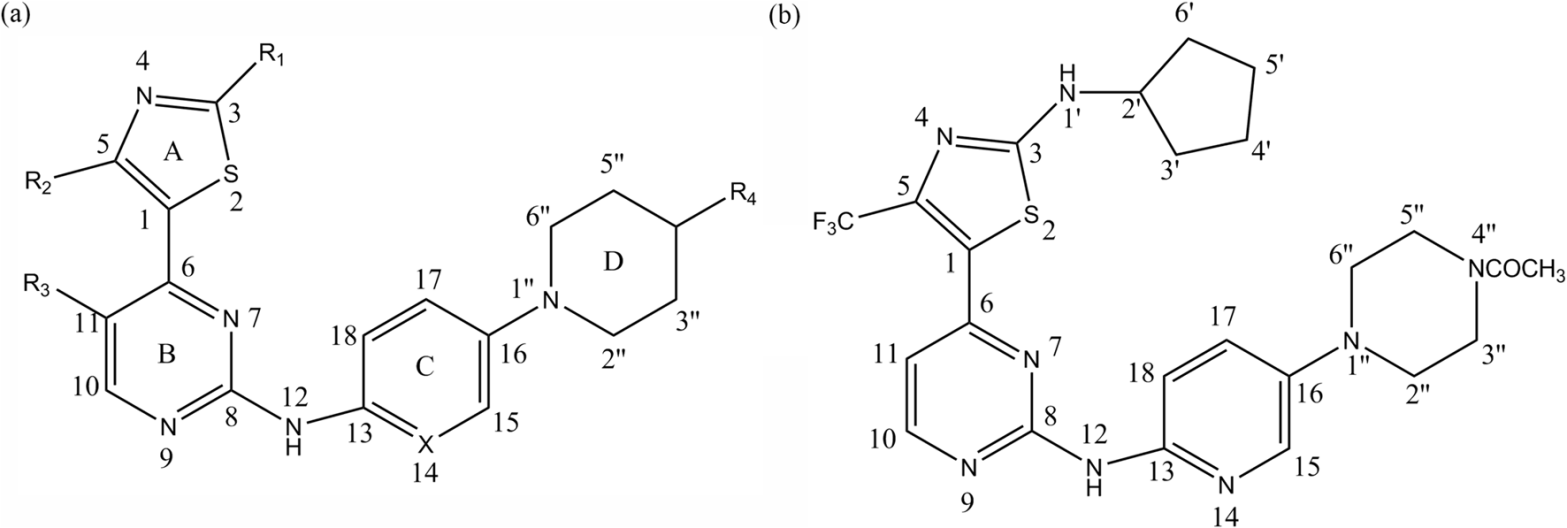
General structural formulas and numbering of benzamide-based derivatives (**a**) and the template molecule (**b**), compound **19**

The key structural forms of the investigated compounds are shown in Fig. 1a. The total set of these derivatives was divided into a training set (54 compounds) and a test set (18 compounds) (Table 3). The test compounds were selected manually based on the structural diversity and breadth of their activities. The IC_50_ values for CDK2, CDK4 and CDK6 were converted to pIC_50_ (−log IC_50_) values and utilized as dependent variables for the 3D-QSAR analyses.

The 3D structures of 4-substituted N-phenylpyrimidin-2-amine derivatives were constructed through the sketch molecule module in SYBYL 6.9 software.

A Powell gradient algorithm and Tripos force field were used to calculate the minimal structural energy with a convergence criterion of 0.001 kcal/(mol-A) and as many as 1000 iterations. An MMFF94 charge was assigned to each compound. The minimized structure was used as the initial conformation for molecular docking.

### Molecular docking

To ascertain possible binding consistencies and directions of the interactions between the investigated compounds and CDK2, CDK4 and CDK6, molecular docking was performed utilizing the Surflex-Dock module in SYBYL 6.9 software. The crystal structures of CDK2, CDK4 and CDK6 were obtained from the RCSB protein database (CDK2 PDB ID: 2WIH; CDK4PBD ID: 2W96; and CDK6PBD ID: 2EUF) and used in successive docking experiments without minimizing the energy.

The ligands were docked at the binding sites of the relevant proteins by an empirical scoring function and the patented Surflex-Dock search engine (Paggi et al. 2024). Before docking, we removed all the native ligands and water molecules and added polar hydrogen atoms to the corresponding ligands. Additionally, Kollman-all atom charges were allocated to the protein atoms.

During this research, self-docking was used to generate ProtoMol, which has two important parameters: the ProtoMol-bloat and the ProtoMol-threshold were used at the default values of 0 and 0.50 Å, respectively.

When other parameters were set to their default values, Surflex-Dock produced 20 conformations each ligand. Subsequently, the binding conformation with the highest docking score for the cocrystallized ligands under similar circumstances was chosen for use in the subsequent 3D-QSAR research. During the whole process, the ligand compound became increasingly flexible, and the protein was defined as a stiff species.

### Molecular modeling and alignment

When operating on an SGI R2400 platform, an additional package of the SYBYL 6.9 molecular modeling program produced CoMFA models, with all the parameters set as the default values unless otherwise described.

The structural comparison rules of these compounds are pivotal for the CoMFA analysis (Yu et al. 2023).

To acquire the best 3D-QSAR model, two disparate alignment methods were used. The first was a ligand-based alignment; that is, all the compounds were compared against the most active compound **19**, in the Align Database command in SYBYL 6.9 software. The other method is based on receptor alignment.

During this process, the final conformations of these compounds in the docking procedure were allocated MMFF94 charges and were arranged for the molecular comparison by 3D-QSAR analysis. The basic structure and the compounds identified above are shown in Fig. 1.

### Generation of 3D-QSAR models

In the CoMFA analytical process, the spatial and electrostatic field models are based on Lennard–Jones and Coulombic potential.

To calculate space and electrostatic energy, we used the Tripos force field, which has a distance-dependent dielectric constant at all intersections in a regularly spaced (2 Å) grid with sp carbon atoms. CoMFA can effectively determine spatial and electrostatic mutual effects. In addition, it also easily calculates the corresponding fields by the proper usage of a sp^3^ carbon probe atom with a charge of + 1.0 and a van der Waals radius of 1.0 Å. The Gaussian functional form is used for the calculation between probe atoms and molecular atoms. The default value should be set to 0.3.

### Partial least squares (PLS) analysis and validation of the QSAR models using the partial least-square (PLS) statistical method

The arithmetic-partial least-square (PLS) statistical method is commonly used for 3D-QSAR equations [ (Chatterjee et al. 2023; Chatterjee and Roy 2023)]. Based on the partial least-square method, the leave-one-out (LOO) cross-validation method was applied to acquire the supreme cross-validation correlation coefficient and the most appropriate quantitative value of elements N.

We then performed the non-cross validation method and calculated the customary correlation coefficient R^2^, the standard error of estimation (SEE) and the F value. To further evaluate the robustness and statistical validity of the derived model, a 100-run bootstrap analysis was also performed.

The non-cross-validation methods were then used, and the conventional correlation coefficient R^2^, standard error of estimates (SEE), and F value were calculated. To assess robustness and statistical effectiveness of the models, we performed 100 runs in a bootstrapping analysis.

To evaluate the forecasting ability of the 3D-QSAR model, which is based on the training set, the biological activity of a test set of 18 compounds was used.

The estimated ability of the patterns is evaluated by the predictive correlation coefficient (R^2^_pred_) computed using the equation: R^2^_pred_ = (SD-PRESS)/SD. SD is the sum of the squared deviations of the biological activities of the test set compounds to the mean activity of the training set compounds, while PRESS is the numerical value of squared deviations of the discrepancies between real values and predicted values calculated with the test set compounds.

As indicated by Golbraikh and Tropsha and their associates (Golbraikh and Tropsha 2002), the 3D-QSAR model is effective if all of the following conditions are satisfied: q^2^ > 0.5, R^2^ > 0.6 and R^2^_*pred*_ > 0.6.

### Molecular dynamics (MD) simulations

We utilized AMBER 9 software for the MD simulation to further verify our results (Case et al. 2005). We regarded the 2WIH, 2W96 and 2EUF chemical compounds and the **3**, **4**, **19**, **31**, **57**, **71** and **72** compounds as our initial docking structures. Our laboratory calculated the electrostatic potential (ESP) of each compound at the B3LYP/6-31G(d) level in the Gaussian 09 program and applied the RESP protocol to produce a portion of the atomic charges of these compounds. To further modify the protein, we ran this program in AMBER FF03, while using the general AMBER force field (gaff) as the ligand. Because of our previous experience, we applied Na+ as a counter ion of to neutralize the compound, and then, the TIP3P water model was utilized to immerse the whole system, extending a margin of 12 Å.

Before we started the molecular dynamics simulation, a two-stage energy minimization was required to relieve possible stress. In the initial stage, the overall system was constrained by 2.0 kcal/(mol·Å^2^), and water molecules and Na+ ions were minimized through the steepest descent of 2000 steps and a conjugate gradient of 3000 steps. In the following phase, the entire system was gradually set to a minimum of 5000 steps in the steepest descent method following a 5000-step conjugated gradient method.

Then, each complex of the system was increasingly heated from 0 to 300 K at a constant volume for less than 200 ps and equilibrated at 1 atm and 300 K for 500 ps.

The simulation lasting for 50 ns for every complex in an NPT (constant number of molecules, p = 1.0 atm and T = 300 K) ensemble. In the molecular dynamics simulation, we evaluated the long-range electrostatic interactions using a nonbonded cutoff of 8.0 Å, while overall restraining the covalent bonds through hydrogen atoms using the SHAKE algorithm with 2 fs as the time for every step. To evaluate empirically the coordinate trajectories, which were reported by Amber software for every 1 ps, we applied the root mean square deviation (RMSD) methodology to the calculation. We were then equipped to determine the integral level of each compound by analyzing their trajectory parameters.

### Binding free energy calculations

With the purpose of verifying the binding stabilization of the complexes described above, we employed the MM-PBSA program in AMBER 9 to compute the binding free energy as

$$ \begin{aligned} \Delta {\text{G}}_{{{\text{bind}}}} & = {\text{G}}_{{{\text{complex}}}} - \left( {{\text{G}}_{{{\text{receptor}}}} + {\text{G}}_{{{\text{ligand}}}} } \right) \\ & = \Delta {\text{E}}_{{{\text{MM}}}} + \Delta {\text{G}}_{{{\text{sol}}}} - {\text{T}}\Delta {\text{S}} \\ & = \Delta {\text{E}}_{{{\text{vdw}}}} + \Delta {\text{E}}_{{{\text{ele}}}} + \Delta {\text{G}}_{{{\text{ele}},{\text{sol}}}} + \Delta {\text{G}}_{{{\text{nonpol}},{\text{solol}},{\text{sol}}}} - {\text{T}}\Delta {\text{S}} \\ \end{aligned} $$

In this equation, we define the gas-phase interaction energy as ΔE_MM_, which is equal to the totality of van der Waals energy ΔE_vdw_ plus ΔE_ele_, representing electrostatic energy. The ΔGsol represents the solvation free energy, which is determined by polar solvation free energy, called ΔG_ele,sol_, as well as ΔG_nonpol,sol_, the nonpolar solvation free energy. Both the polar solvation free energy (ΔG_ele,sol_) and the nonpolar solvation free energy (ΔG_nonpol,sol_) were evaluated by both the generalized Born approximation model and the solvent-accessible surface area, calculated by AMBER 9. Eventually, through utilizing the MM-GBSA method, the free energy of binding was broken down by each residue, without calculating the entropy contribution (TΔS) due to a lack of scientific research funds.

The ΔG_ele,sol_ and ΔG_nonpol,sol_ were assessed by the generalized Born (GB) approximation model and the solvent-accessible surface area (SASA), which was computed with the MolSurf module in AMBER 9. Due to the expensive computational demand and the lack of satisfactory results, the TΔS was not evaluated in this study. Finally, the binding free energies were decomposed to each residue by the MM-GBSA method.

## Results

### Docking results

**Fig. 2:**
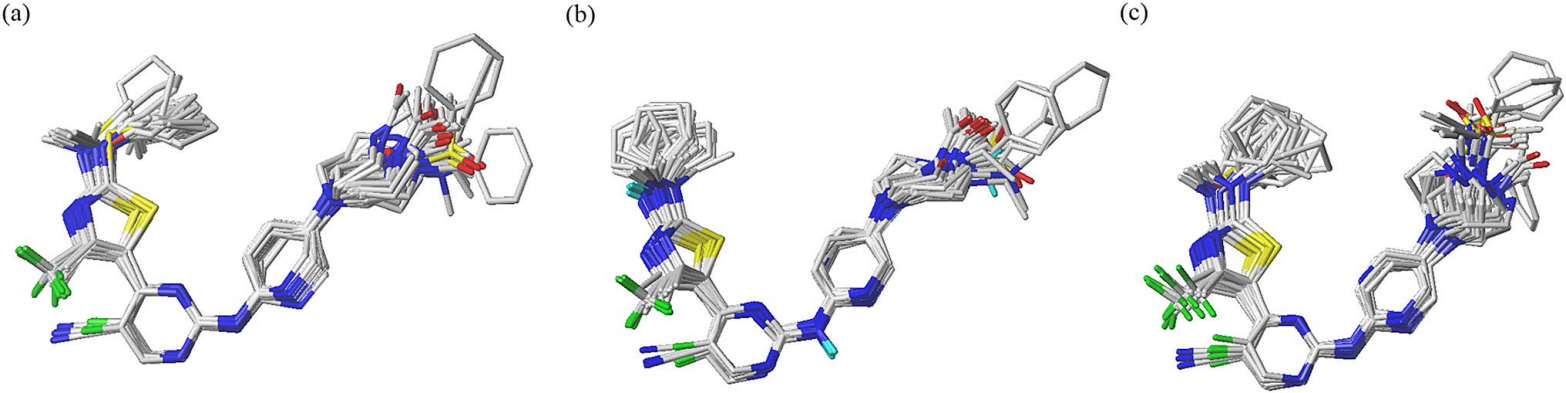
Superposition of the 72 studied compounds, **a** CDK2, **b** CDK4, and **c** CDK6

**Fig. 3:**
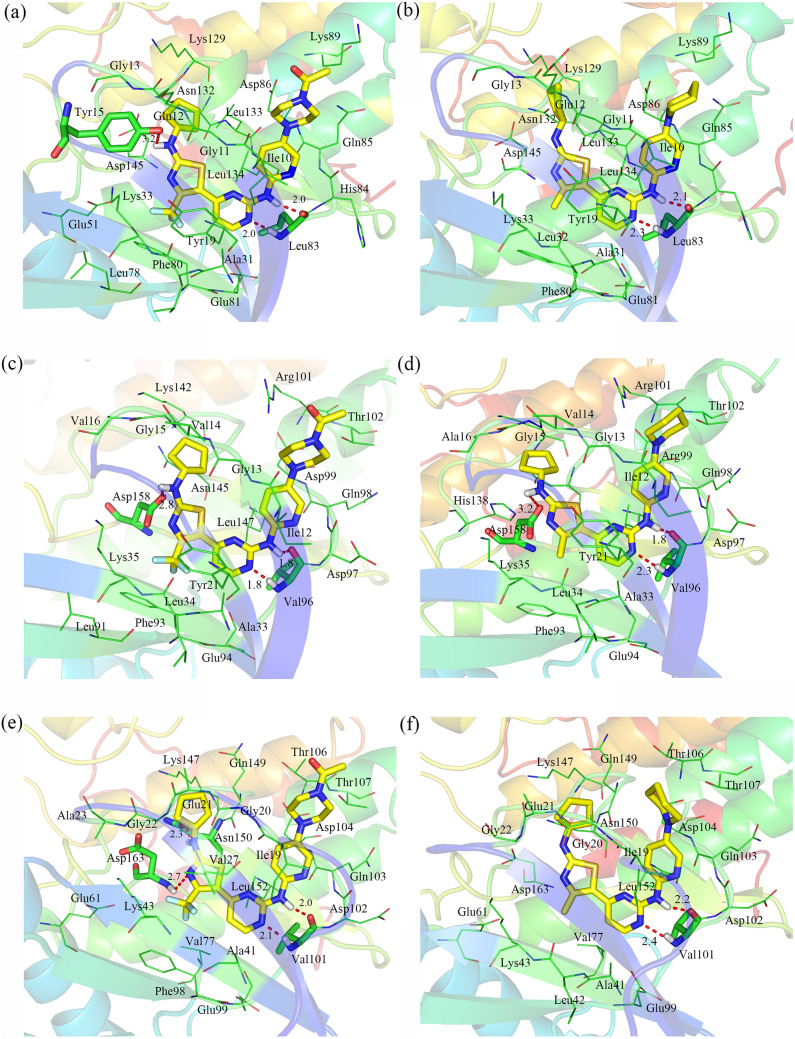
Docking models of the highly potent compound **19** (yellow) in the binding sites of CDK2 (**a**), CDK4 (**c**) and CDK6 (**e**) and that of the less potent compound **72** (yellow) in the binding sites of CDK2 (**b**), CDK4 (**d**), and CDK6 (**f**). Hydrogen bonds are depicted as red dotted lines

All 72 studied inhibitors docked adequately into the binding pockets of CDK2/4/6, and their optimal conformations were superimposed on each other, as shown in Fig. 2. We chose compounds **19** and **72** as representative for the sake of the discussion. Compound **19** had relatively high activity, and its binding site with CDK2/4/6 adequately accounts for the mechanism between ligands and receptors. Compared with compound **19**, compound **72** showed an obvious decrease in activity with CDK2/4/6 kinases due to the different groups on the R_2_ and R_4_ substituents of compound 72. To further explain why the activity was decreased for compound **72**, we conducted a detailed analysis on the binding modes of compounds **19** and **72**, and their binding patterns when docked into CDK2/4/6 kinases were determined, as demonstrated in Fig. 3.

#### For CDK2 kinase

As shown in Fig. 3a and b, compounds **19** and **72** were adequately docked into the ATP-binding sites of the CDK2 kinase with similar binding modes. In the binding models, the N_9_ atom at the pyrimidine ring and the linker amino (N_12_H) formed two hydrogen bonds with Leu83, with the corresponding distances of 2.0 Å and 2.0 Å for **19** and 2.1 Å and 2.3 Å for **72**. Additionally, compound 19 also formed a hydrogen bond with Tyr15, demonstrating that the hydrogen bond interactions were stronger in the CDK2-**19** complex than they were in the CDK2-**72** complex. The cyclopentyl ring of the R_1_ substituent penetrated into the hydrophilic pocket created by Gly11, Glu12, Gly13 and Leu133, suggesting that hydrophobic substituents at this position increase activity (compounds **1**–**2**). In addition, the R_2_ substituent was near a hydrophilic pocket surrounded by Lys33, Glu51 and Asp145, where strong electrostatic interactions readily occurred when R_2_ held electronegative groups. Thus, for the complex CDK2-19 (R_2_ = CF_3_), the electrostatic interaction was markedly stronger than that for CDK2-72 (R_2_ = CH_3_), which might explain the decrease in compound **72** bioactivity. R_3_ was embedded in a small hydrophobic pocket made up of residues Tyr19, Ala31, Leu32 and Phe80, which were, in some instances, far from the two compounds. Obviously, introducing a moderate-sized group at this position was favorable for the activity. Moreover, the pyrimidine rings of both compounds formed an aromatic-aromatic interaction with the phenyl moiety of Tyr19. The residues His84 and Gln85 appeared close to the X-position of the pyridine ring, illustrating that a hydrophilic atom was favorable for bioactivity at this position. However, apart from His 84 and Gln85, some hydrophobic residues, i.e., Ile10, Leu83 and Leu134, were short distances from the X-position of the pyridine ring, which revealed that introducing a hydrophobic atom at the X-position or substituting a pyridine ring with a benzene ring increased bioactivity. By comparing the bioactivity of compounds 31 with 71, we found that 31 (X = C) clearly exhibited bioactivity that was more than 100-fold greater than that of 71 (X = N). This result suggests that replacing the pyridine ring with a benzene ring is advantageous for augmenting bioactivity. Finally, the R_4_ substituent was extended into the solvent-accessible region at the entrance of the binding pocket encompassed by Gln85, Asp86 and Lys89, in which N_4’’_ atoms of compound 19 could be easily protonated, resulting in strong electrostatic interactions with the negatively charged side chains of Gln85, Asp86 and Lys89. In contrast with that of compound 19, the R_4_ substituent of compound 72 was replaced by a CH_2_ group, whose hydrophilic property was apparently weaker, and the electrostatic interactions between surrounding residues was also weaker, which therefore led to lower potency compared with that of compound 19.

#### For CDK4 kinase

Figure 3c and d indicates the binding modes of compound **19** and **72** with CDK4. Both compounds formed three hydrogen bonds with the hinge region: one between the N_1’_H group of the R_1_ substituent ring and Asp158 and the other two between the N_9_ of the pyrimidine ring and the N_12_H groups and Val96, with the approximate distances of 1.8 Å, 1.8 Å, and 2.0 Å for **19** and 1.8 Å, 2.3 Å and 3.2 Å for **72**. Due to the shorter hydrogen bond, compound 19 might form stronger hydrogen bond interactions than compound **72**. In addition to the hydrogen bond interactions, the cyclopentyl moiety of the R_1_ substituent established van der Waals interactions with the side chains of Gly13, Val14, Gly15 and Val16, where an overlarge group can easily collided and cause strong repulsive interactions. This unfavorable interaction with plasma enzymes caused an obvious decrease in CDK2 bioactivity (compound **11**). In addition, the R_2_ substituents of both compounds suitably embedded in a hydrophilic pocket including Tyr21, Lys33 and Asp158, and these residues formed the strong electrostatic interactions with compound **19** (R_2_ = CF_3_) but not with compound 72 (R_2_ = CH_3_). The R_3_ substituent was buried in a small hydrophobic pocket surrounded by Tyr21, Ala33, Leu34 and Phe93, which provided sufficient space to accommodate relatively large groups at this position. In addition, the pyrimidine ring exhibited an aromatic-aromatic interaction with the phenyl moiety of Tyr21. At the N_14_-position of the pyridine ring, Ile12 and Leu147 tended to make contact with the benzene ring through hydrophobic interactions. Therefore, we can infer that a carbon atom in the X-position was beneficial for activity, which might be the reason why compound 19 did not show the highest bioactivity. Finally, we observed that Asp99, Arg101 and Thr102 constituted an charged region at the entrance of the binding pocket. These residues form electronegative interactions with the protonated N_4’’_ atom of compound **19** but not with compound **72**. In summary, all of these analyses revealed that electrostatic interactions, hydrophobic interactions and repulsive interactions may be critical for the different activity levels of compounds **19** and **72**.

#### For CDK6 kinase

As shown in Fig. 2e and f, the N_9_-position of the pyrimidine ring and linker amino (N_12_H group) formed two hydrogen bonds with Val101, with the corresponding distances of 2.0 Å, 2.1 Å for **19** and 2.2 Å, 2.4 Å for **72**. In addition to these hydrogen bonds, compound 19 gained another two hydrogen bonds at the hinge region: one with Asn150 (Asn150 = O…NH, bond length of 2.3 Å) and the other one with Asp163 (Asp163-NH…N, bond length of 2.7 Å). The cyclopentyl moiety of the R_1_ substituent lay close to the side chains of Ala23, Gly22, Glu21 and Gly20 and contacted these residues in van der Waals interactions. In addition, the CF_3_ group of compound 19 was readily protonated, which probably enabled strong electrostatic interactions with surrounding residues (Lys43 and Asp163). In contrast with that of compound **19**, the R_2_ substituent of compound **72** was replaced by a methyl group and forming negligible electrostatic interactions with Lys43 and Asp163. We also found that the basic nitrogen atom at the pyridine ring was in the vicinity of Ile19 and Leu152, thus favorably implanting a carbon atom at the **X**-position. We also observed a hydrophilic pocket near the entrance of the binding pocket. This pocket involved Asp104, Thr106 and Thr107, all polar residues that readily formed electrostatic interactions with the protonated atom (N_4’’_ atom) of the R_4_ substituent in compound **19** but not with compound **72**. Regarding compound 72, two reasons explain the dramatic loss of activity. One is a lack of protonated atoms at the R_2_ and R_4_ substituents, and the other one is the absence of corresponding hydrogen bonds (Asn150 = O…NH and Asp163-NH…N).

#### Comparison of CDK2/4/6 kinases

**Fig. 4:**
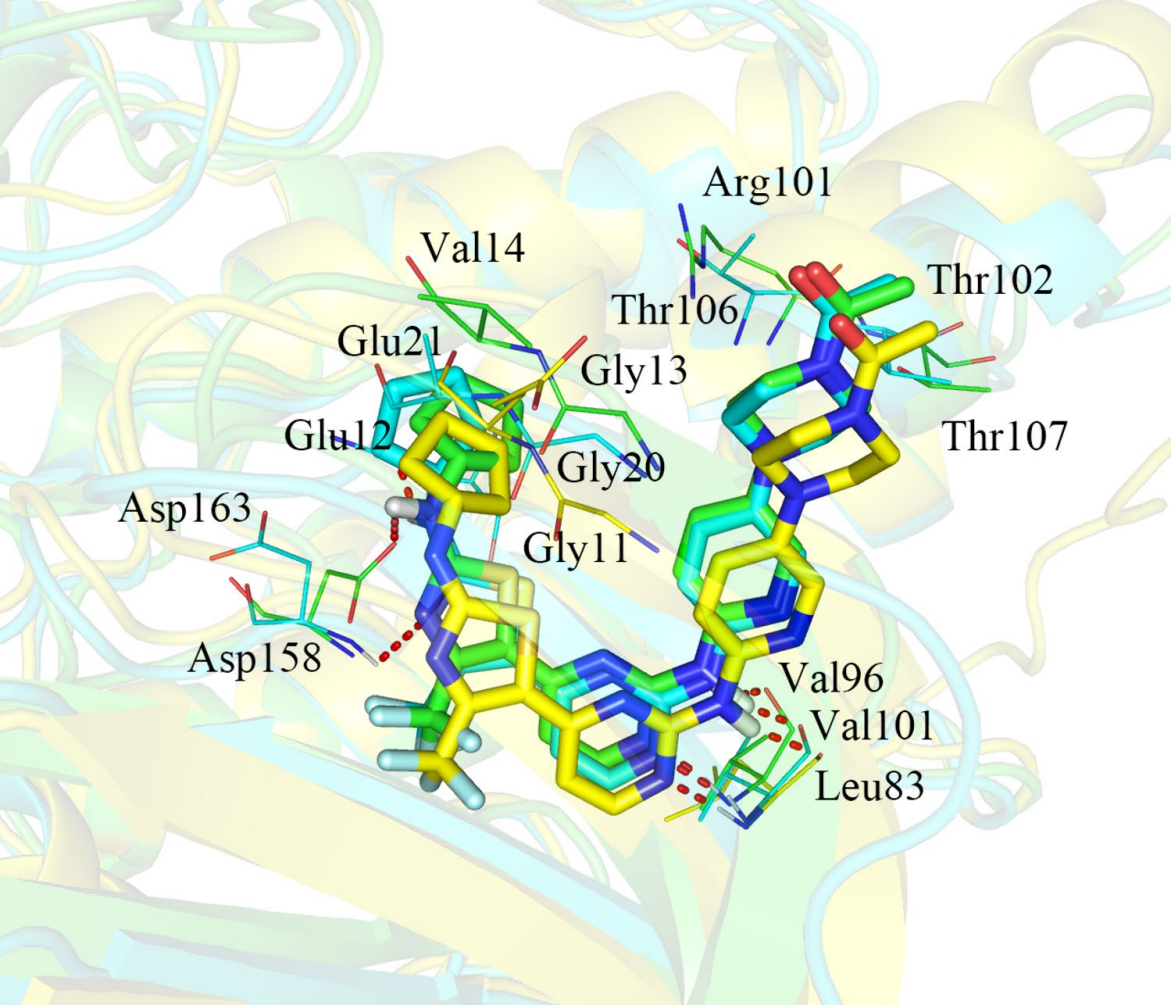
Structural comparison of CDK2-**19** (yellow), CDK4-**19** (green) and CDK6-**19** (cyan)

As shown in Fig. 4, we found that the binding sites of compound **19** to CDK2/4/6 were nearly identical, but some differences were observed. First, the cyclopentyl moiety was located in the shadow of Val14 in CDK4 with favorable hydrophobic interactions, whereas no such interactions were formed with Glu12/Glu21 of CDK2/CDK6. Second, the protonated N_4’’_ atoms situated close to the negatively charged side chain of Arg101 in CDK4, exhibiting a stronger electrostatic interaction than Lys89/Thr106 of CDK2/CDK6. Third, compound **19** formed three hydrogen bonds for CDK2 (bond lengths of 2.0, 2.0 and 3.2 Å), three hydrogen bonds for CDK4 (bond lengths of 1.8, 1.8 and 2.8 Å) and four hydrogen bonds for CDK6 (bond lengths of 2.0, 2.1, 2.3 and 2.7 Å), suggesting that the hydrogen bond interactions became slightly stronger in the CDK6-**19** complex than in CDK2-**19** and CDK4-**19** complexes. Nonetheless, the increase in the number of hydrogen bond interactions in the CDK6-**19** complex did not counteract the impairment of hydrophobic and electrostatic interactions, leading to the lower activity of **19** for CDK6 than CDK4.

#### CoMFA statistical analysis results

**Fig. 5:**
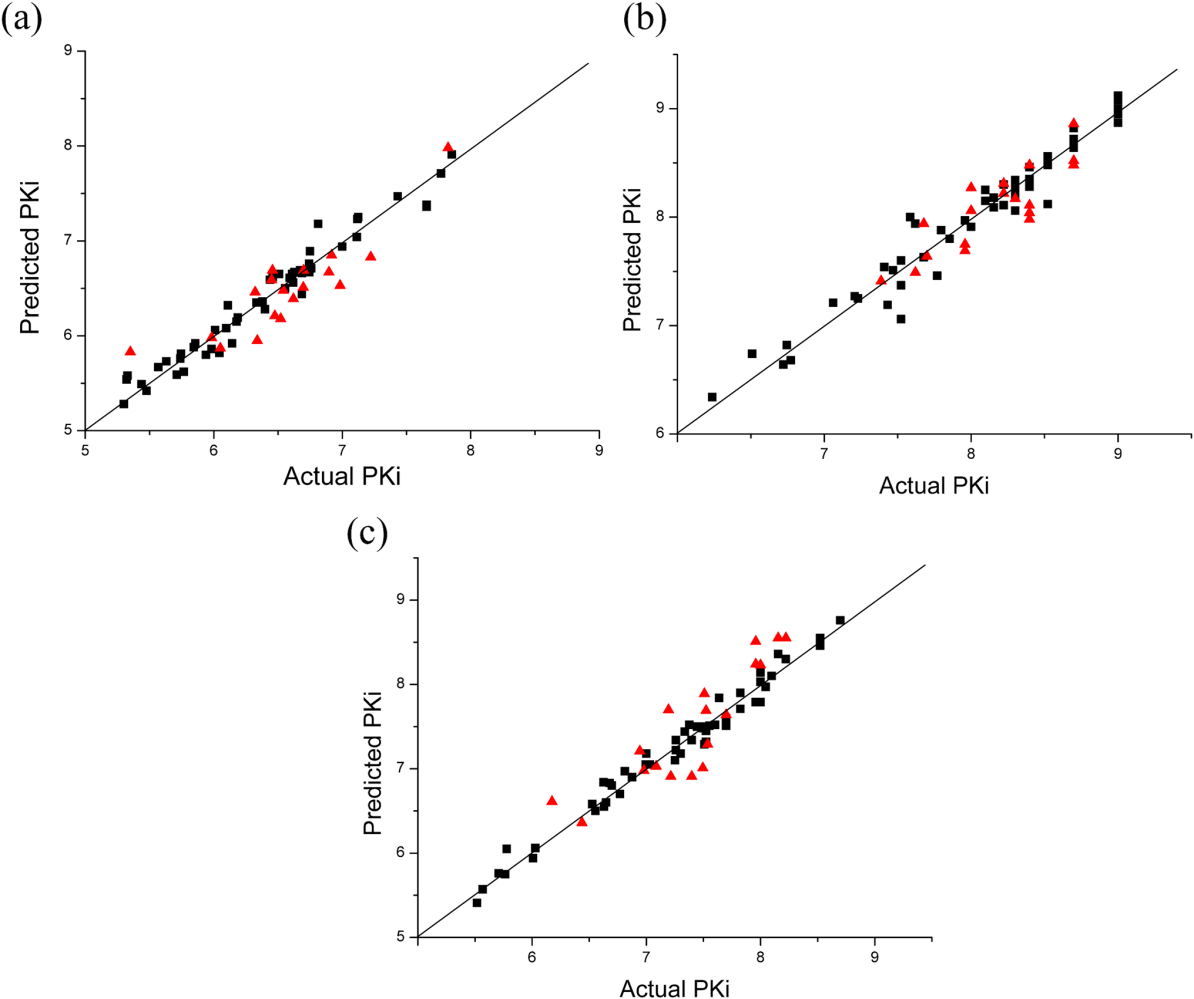
Scatter plots of predicted activities vs. actual activities using the training set (black square) and test set (red triangle) for the CoMFA models of CDK2 (**a**), CDK4 (**b**) and CDK6 (**c**)

The CoMFA analyses were utilized to create the 3D-QSAR models of CDK2, CDK4 and CDK6 kinases, and the detailed statistical parameters are represented in Table 2. Depending on the training set (54 compounds) and the test set (18 compounds), all three models exhibited good correlation coefficients (*q*^2^, *R*^2^, *F* and *SEE*), with 0.714, 0.962, 179.575 and 0.139 for CDK2; 0.815, 0.976, 313.343 and 0.116 for CDK4; and 0.757, 0.977, 330.996 and 0.125 for CDK6, respectively. The bootstrapping results (100 runs) showed *R*_*bs*_^2^ and *SD*_*bs*_ values of 0.973 and 0.01 for CDK2, 0.985 and 0.006 for CDK4, 0.985 and 0.005 for CDK6, respectively, demonstrating that these models had satisfactory internal predictability. Moreover, the predictive correlation coefficients *R*^2^_pred_ for the three test sets were 0.762, 0.681 and 0.674, respectively, revealing that the three models had reliable external predictability. Comparing the contributions of steric and electrostatic fields, we discovered that the electrostatic fields of the three models all exerted higher influences than steric fields on their inhibitory activity, as shown in Table 2. The predicted pIC_50_ values of the three established CoMFA models are listed, and the residual values with experimental pIC_50_ were computed, as shown in Table 3. We clearly observed that the experimental pIC_50_ values were in line with the predicted *pIC*_*50*_ values in Fig. 5, in which most points were equally distributed around the regression line, verifying good reliabilities of the three obtained CoMFA models.

**Table 2:** Statistical analysis results of the CoMFA models^a^

	CDK2	CDK4	CDK6
PLS statistic			
*q*^*2*^	0.714	0.815	0.757
*R*^*2*^	0.962	0.976	0.977
*F*	179.575	313.343	330.996
*SEE*	0.139	0.116	0.125
*R*_*bs*_^*2*^	0.973	0.985	0.985
*SD*_*bs*_	0.01	0.006	0.005
*R*^*2*^_*pred*_	0.762	0.681	0.674
Field distribution %			
*S*	0.423	0.407	0.443
*E*	0.577	0.593	0.557

^a^*N* is the optimal number of components, *q*^2^ is the square of LOO cross-validation coefficient, *R*^2^ is the square of non-cross-validation coefficient, *SEE* is the standard error of estimation, *F* is the *F*-test value, *R*_bs_^2^ is the mean *R*^2^ of bootstrapping analysis (100 runs), *SD*_bs_ is the mean standard deviation by bootstrapping analysis

**Table 3:** The total set of these derivatives was divided into a training set (54 compounds) and a test set (18 compounds)

No.	CDK2			CDK4			CDK6		
	Expt. pKi	Pred.pKi	Res	Expt. pKi	Pred.pKi	Res	Expt. pKi	Pred.pKi	Res
				Training set					
1	6.56	6.50	0.06	9.00	9.08	− 0.08	8.10	8.10	0.00
2	6.69	6.66	0.03	8.70	8.64	0.06	8.05	7.97	0.08
4	6.11	6.32	− 0.21	8.22	8.30	− 0.08	7.03	7.05	− 0.02
5	5.33	5.58	− 0.25	8.40	8.28	0.12	7.52	7.32	0.20
6	6.74	6.76	− 0.02	8.52	8.48	0.04	6.88	6.90	− 0.02
7	7.11	7.04	0.07	8.15	8.09	0.06	7.26	7.34	− 0.08
8	6.76	6.71	0.05	8.70	8.68	0.02	8.00	8.14	− 0.14
11	5.98	5.86	0.12	7.59	8.00	− 0.41	7.00	7.18	− 0.18
13	6.10	6.08	0.02	7.55	7.88	− 0.33	7.55	7.51	0.04
14	6.40	6.28	0.12	7.82	8.12	− 0.30	7.82	7.71	0.11
17	7.66	7.38	0.28	8.10	8.15	− 0.05	8.70	8.76	− 0.06
18	7.66	7.36	0.30	9.00	8.87	0.13	8.52	8.46	0.06
19	7.85	7.91	− 0.06	8.40	8.35	0.05	8.22	8.30	− 0.08
20	6.81	7.18	− 0.37	8.10	8.25	− 0.15	7.96	7.79	0.17
22	6.59	6.61	− 0.02	8.52	8.56	− 0.04	8.15	8.36	− 0.21
23	6.38	6.36	0.02	7.85	7.80	0.05	8.00	8.03	− 0.03
24	6.99	6.98	0.01	9.00	8.97	0.03	8.52	8.55	− 0.03
26	6.64	6.67	− 0.03	7.47	7.51	− 0.04	7.64	7.84	− 0.20
28	6.46	6.62	− 0.16	7.41	7.54	− 0.13	7.00	7.05	− 0.05
29	5.94	5.80	0.14	9.00	8.99	0.01	7.51	7.29	0.22
30	7.77	7.71	0.06	9.00	9.00	0.00	7.44	7.50	− 0.06
31	7.43	7.47	− 0.04	8.22	8.11	0.11	6.65	6.60	0.05
33	6.19	6.19	− 0.00	8.30	8.34	− 0.04	7.30	7.18	0.12
34	6.51	6.65	− 0.14	6.24	6.34	− 0.10	5.52	5.41	0.11
36	6.67	6.69	− 0.02	9.00	9.12	− 0.12	7.40	7.34	0.06
40	6.63	6.67	− 0.04	8.40	8.46	− 0.06	7.49	7.50	− 0.01
41	6.44	6.59	− 0.15	8.70	8.67	0.03	8.00	7.79	0.21
42	6.18	6.15	0.03	7.70	8.06	− 0.36	7.70	7.60	0.10
43	6.75	6.89	− 0.14	7.77	7.46	0.31	7.34	7.44	− 0.10
44	7.00	6.94	0.06	8.30	8.19	0.11	7.52	7.45	0.07
45	7.12	7.23	− 0.11	7.43	7.19	0.24	6.53	6.58	− 0.05
46	5.48	5.42	0.06	8.70	8.72	− 0.02	6.55	6.50	0.05
47	6.69	6.44	0.25	8.52	8.54	− 0.02	7.49	7.48	0.01
49	5.86	5.92	− 0.06	8.70	8.82	− 0.12	7.26	7.22	0.04
51	6.04	5.82	0.22	7.68	7.63	0.05	7.25	7.10	0.15
52	5.84	5.88	− 0.04	7.52	7.37	0.15	6.81	6.97	− 0.16
53	6.01	6.06	− 0.05	7.06	7.21	− 0.15	6.63	6.55	0.08
54	5.71	5.59	0.12	6.77	7.06	− 0.29	6.77	6.70	0.07
55	5.77	5.62	0.15	7.23	7.25	− 0.02	6.63	6.84	− 0.21
56	5.74	5.76	− 0.02	7.62	7.94	− 0.32	6.01	5.94	0.07
57	5.74	5.81	− 0.07	8.00	7.91	0.09	5.78	6.05	− 0.27
58	5.63	5.73	− 0.10	6.74	6.82	− 0.08	5.76	5.75	0.01
59	6.62	6.56	0.06	7.96	7.97	− 0.01	7.52	7.48	0.04
61	6.33	6.35	− 0.02	8.30	8.26	0.04	7.60	7.52	0.08
62	6.61	6.65	− 0.04	7.21	7.27	− 0.06	6.68	6.83	− 0.15
64	5.44	5.49	− 0.05	6.51	6.74	− 0.23	6.03	6.06	− 0.03
65	6.14	5.92	0.22	8.30	8.25	0.05	7.70	7.55	0.15
66	5.57	5.67	− 0.10	8.15	8.18	− 0.03	7.38	7.52	− 0.14
67	5.32	5.54	− 0.22	6.72	6.64	0.08	5.71	5.76	− 0.05
68	6.74	6.67	0.07	7.52	7.60	− 0.08	6.70	6.80	− 0.10
69	6.74	6.71	0.03	9.00	8.95	0.05	7.82	7.90	− 0.08
70	7.12	7.25	− 0.13	8.30	8.28	0.02	7.70	7.51	0.19
71	5.30	5.28	0.02	6.77	6.68	0.09	5.57	5.57	0.00
72	5.30	5.41	− 0.11	7.16	7.22	− 0.06	6.59	6.77	− 0.18
				Test set					
3	6.45	6.59	− 0.14	8.67	8.52	0.15	7.96	8.24	− 0.28
9	6.32	6.46	− 0.14	8.70	8.86	− 0.16	8.00	8.23	− 0.23
10	6.92	6.85	0.07	8.00	8.27	− 0.27	7.51	7.89	− 0.38
12	6.62	6.39	0.23	7.96	8.17	− 0.21	7.96	8.51	− 0.55
15	6.70	6.69	0.01	8.15	7.94	0.22	6.98	6.98	0.00
16	6.90	6.67	0.23	7.68	7.41	0.27	7.09	7.03	0.06
21	7.82	7.98	− 0.16	7.39	7.69	− 0.30	8.15	8.55	− 0.40
25	6.46	6.69	− 0.23	8.70	8.48	0.22	8.22	8.12	0.10
27	6.98	6.53	0.46	8.22	8.31	− 0.09	7.70	7.64	0.06
32	6.05	5.87	0.18	8.22	8.22	0.00	6.94	7.21	− 0.27
35	6.34	5.95	0.39	7.70	7.64	0.06	7.21	6.91	0.30
37	6.52	6.18	0.34	8.40	8.48	− 0.08	7.49	7.01	0.48
38	5.35	5.83	− 0.48	8.40	8.04	0.36	7.52	7.69	− 0.17
39	6.70	6.51	0.18	8.40	8.11	0.29	7.19	7.70	− 0.51
48	6.47	6.21	0.26	8.40	7.98	0.42	7.40	6.91	0.49
50	5.98	5.98	0.00	7.62	7.49	0.13	6.44	6.36	0.08
60	6.54	6.48	0.06	8.00	8.06	− 0.06	7.54	7.29	− 0.25
63	7.22	6.83	0.39	7.96	7.75	0.21	6.17	6.61	− 0.44

### CoMFA contour map for CDK2

**Fig. 6:**
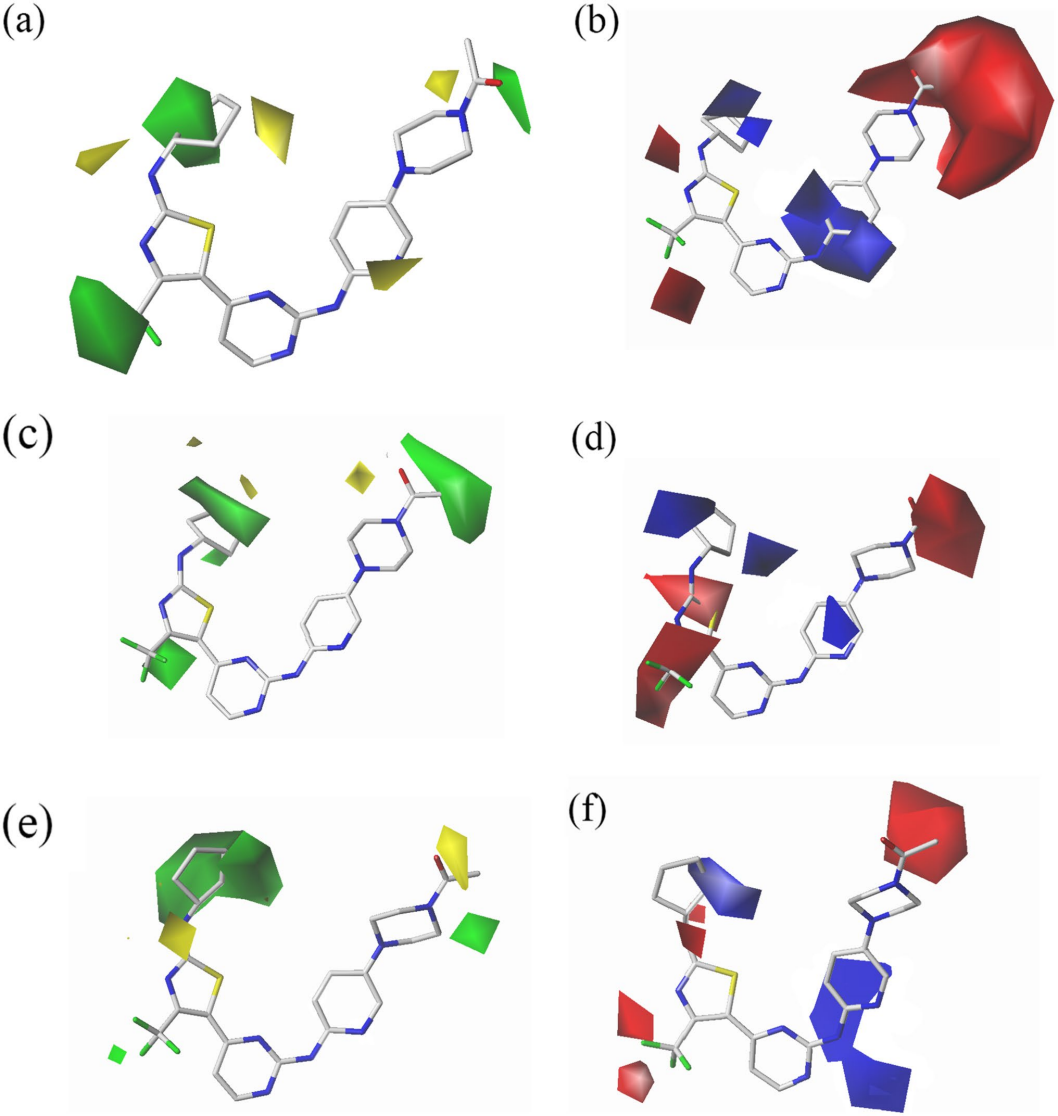
CoMFA contour maps of the highly potent compound **19**. Steric contours for CDK2 (**a**), CDK4 (**c**) and CDK6 (**e**). Green contours indicate regions where bulky groups increase activity, while yellow contours indicate regions where bulky groups decrease activity. Electrostatic contours for CDK2 (**b**), CDK4 (**d**) and CDK6 (**f**). Blue contours indicate regions where positive charges improve activity; red contours indicate regions where charges improve activity

For each enzyme, the best CoMFA models were chosen to build the 3D coefficient contour maps to observe the field effects on the target features and thus may be helpful for identifying possible interaction sites. The CoMFA steric contour maps are displayed in Fig. 6a using compound **19** as a reference structure for explanation. The green block represents the steric favorable regions, while the yellow block represents unfavorable regions. In the vicinity of ring E, there are a large green and two small yellow blocks, suggesting that a moderate-sized group is advantageous at this position. This finding was well depicted, with some examples in which compound **2** with cyclopentyl at this position showed higher activities than compounds **14**, **11** or **33** with phenyl, isopropyl and H, respectively. Moreover, there was a bulky green block around trifluoromethyl, which is embedded in a hydrophilic pocket that included Tyr21, Lys33 and Asp158 in the docking results. This finding indicates that the replacement of the R_2_ substituent with a bulky group enhances bioactivity, which can reasonably explain why compounds **18**–**20** with –CF_3_ groups had higher activity levels than compounds **2**, **4** or **5**. A yellow region located above the X-position of ring C demonstrates that introducing a large atomic radius at this position is favorable, which may be a reason why compound **71,** with a nitrogen atom, behaves nearly 150-fold worse than compound **31,** with a carbon atom at the X-position. Thus, there are reasons to believe that a carbon atom at the X-position effectively improves activity. Finally, we also observed a green and yellow region in the proximity of the R_4_ substituent, which is hindered by the side chain of Lys89, revealing that a moderate-sized group is conducive to augmenting bioactivity. However, these docking results (Fig. 2a) verified that the terminal region of the R_4_ substituent extends toward the solvent region. Hence, we speculate that moderate-sized groups of a certain long length were tolerated by the CDK2 kinase.

The contour map of the CoMFA electrostatic field is shown in Fig. 6b. The blue region indicates that electropositive groups were favorable for bioactivity, and the red region indicates that the electronegative groups were beneficial. Five blue blocks were distributed from ring E to the X-position of ring C, which was in line with the hydrophobic side chains of Ile10, Gly11, Gly13, Leu133 and Leu134 in the docking study (Fig. 2a), showing that introducing a high electropositive group in ring E or featuring a carbon atom in the X-position of ring C favorably enhances the bioactivity. This finding can explain why compound **31,** with a carbon atom, showed a bioactivity that was 150-fold greater than that of compound **71,** with a nitrogen atom at the X-position, which suggests that an X-position substituted by a carbon atom might be a significant factor to improve bioactivity. Regrettably, most of the potent compounds, namely, **17**–**21**, retaining the cyclopentyl group in ring E, introduce a nitrogen atom at the X-position instead of a carbon atom. Therefore, we believe that these potent compounds would gain better bioactivity with a carbon atom at the X-position. Furthermore, a red block is shown on the left side of the N_1’_-position, suggesting that the electrogroup was beneficial. The docking result (see Fig. 2a) also revealed that the N_1’_substituent situated near Tyr15 and had the potential to form a hydrogen bond with Asp145. It was verified that compounds **32**, **33** and **35,** with amino groups, were more potent in bioactivity than compounds **55**, **56** and **58,** with methylene groups at this position. Finally, the all of R_4_ was embedded in a large red block, representing Gln85, Asp86 and Lys89 in the docking study (see Fig. 2a), indicating that R_4_ likely introduces groups with strong electronegativity. For example, compound **43,** with a -NCOCH_3_ group, was approximately 20-fold more electronegative than compound **46** with a -CHN(CH_3_)_2_ group. In addition, compound **2,** with higher potency, was also such a case when compared to **72**.

### CoMFA contour map for CDK4

The steric and electrostatic field contours of the CDK4 CoMFA model are clearly displayed in Fig. 6c and d and were roughly similar to those of CDK2. For example, a bulky green and two little yellow contours lying near ring E indicated that a moderate-sized substituent incorporated at this situation exerted a favorable influence on CDK4 bioactivity. However, some differences were also observed between the CDK2 and CDK4 CoMFA models and mainly involved the absence of a yellow block near ring C and the appearance of a big green block between R_2_ and R_3_ in the CDK4 CoMFA model. Moreover, three blue and two red blocks of the CDK4 model were found to be almost the same as those of the CDK2 model, but the sizes of the former were increased. These differences may be critical for the selectivity of CDK2 and CDK4. The appearance of a big green block between R_2_ and R_3_ indicated the need to augment the lengths of R_2_ and R_3_, a finding congruent with the docking results showing that the R_2_ and R_3_ substituents were buried in a bulky pocket surrounded by Tyr21, Ala33, Leu34 Lys35, Leu91, Phe93 and Glu94. This supposition can be rationalized by the examples of compounds **36**–**39,** with -CN groups at the R_2_ position, exhibiting higher bioactivity with CDK4 than compound **32**–**35,** with hydrogen atoms at R_2_. Moreover, three blue blocks distributed from ring E to the X-position of ring C were in accord with the hydrophobic side chains of Ile12, Gly13, Val14, Gly15 and Val16 in the docking study (Fig. 2c), offering an explanation for the marked increase in the CDK4 bioactivity of **33**–**35,** with cyclopentyl groups, compared to **2**, 4 and **5,** with hydrogen atoms. Finally, a red polyhedron engulfing the N_1’_H group suggested that a hydrophilic group incorporated at this position was desirable for enhancing the activity to the CDK4 inhibitor, which was supported by the formation of a hydrogen bond (Asp158 = O…H N_1’_, bond length 2.8 Å) with Asp158 in the docking result (Fig. 2c). Thus, we confirmed that compounds **55**, **56** and **58,** with amino groups, all exhibited nearly tenfold decreases compared to compounds **32**, **33** and **35,** with ethyl groups as R_1_.

### CoMFA contour map for CDK6

The steric field of the CoMFA model is displayed in Fig. 6e and was very similar to that of the CDK2 CoMFA model, except for size differences. Thus, it is not discussed here. Figure 6f represents the electrostatic field and shows some differences compared with CDK2 and CDK4. First, two red blocks situated near the N_1’_H group, while only a single red block is adjacent to this site in CDK2 and CDK4, indicating that the electronegative group featured here was necessary. Many of the potent compounds **1**–**3**, **8**–**9**, **12**, **19**–**21** and **41**–**44** possessed electronegative groups at the N_1’_-position, whereas the most inactive compounds, **57**–**58** and **67,** carried an electropositive group (CH group) at this position. These findings were confirmed by a docking result showing that the N_1’_H group formed a strong hydrogen bond with the near residue Asn150 (bond length 2.3 Å). Second, we observed two red blocks around the R_2_, while only a red block surrounded this region in CDK2 and CDK4. These findings indicated that the electrogroup incorporated in this region was required for a compound to be a potent CDK6 inhibitor. In the docking study, we found Lys43, Glu61 and Asp163 on the left side of the R_2_ substituent forming strong electrostatic interactions with this substituent. Thus, the most potent compounds, 17–**21,** all had -CF_3_ groups rather than -CH_3_ groups. Finally, a large blue block was observed stretching from the N_12_ to the C_16_ position, while just a small blue block was incorporated at the X-position of ring C in CDK4. This finding indicated the need to augment the electropositivity at the X-position, specifying that compound **31,** with a carbon atom, exhibited more than tenfold better activity than **71,** with a nitrogen atom at this location. The docking result (see Fig. 2e) also demonstrated that some residues (Ile19, Val101 and Leu152) with strong electropositivities were in close vicinity to ring **C** and probably established some significant hydrophobic interactions to influence the CDK6 bioactivity.

From the above analysis, we formed some conclusions for CDK2/4/6 kinases:

1. Electrogroups at the N_1_-position and electropositive and moderate-sized groups at ring E may be conducive for bioactivity.
2. Bulky and electrogroups featured at the R_2_ substituent can augment the bioactivity.
3. Selecting a nitrogen atom rather than a carbon atom at the X-position favorably contributed to bioactivity.
4. An Electrogroup at the R_4_ substituent was likely to enhance bioactivity.

### MD simulations, MM/GBSA calculations and free energy decomposition

According to the docking results, we selected seven compounds, **3**, **4**, **19**, **31**, **57**, **71** and **72,** with CDK2, CDK4 and CDK6 kinases to carry out the 50-ns molecular dynamics simulations. These selected compounds were separated into four pairs (3–57, 4–19, 31–71 and 4–72), to specify the influences of different substituent R_1_, R_2_, X and R_4_ groups on CDK2/4/6 bioactivity. To elucidate the dynamic stability and reasonability of these systems, the RMSD values of the protein backbones against the initial structures were computed and are shown in Fig. 5a.

**Fig. 7:**
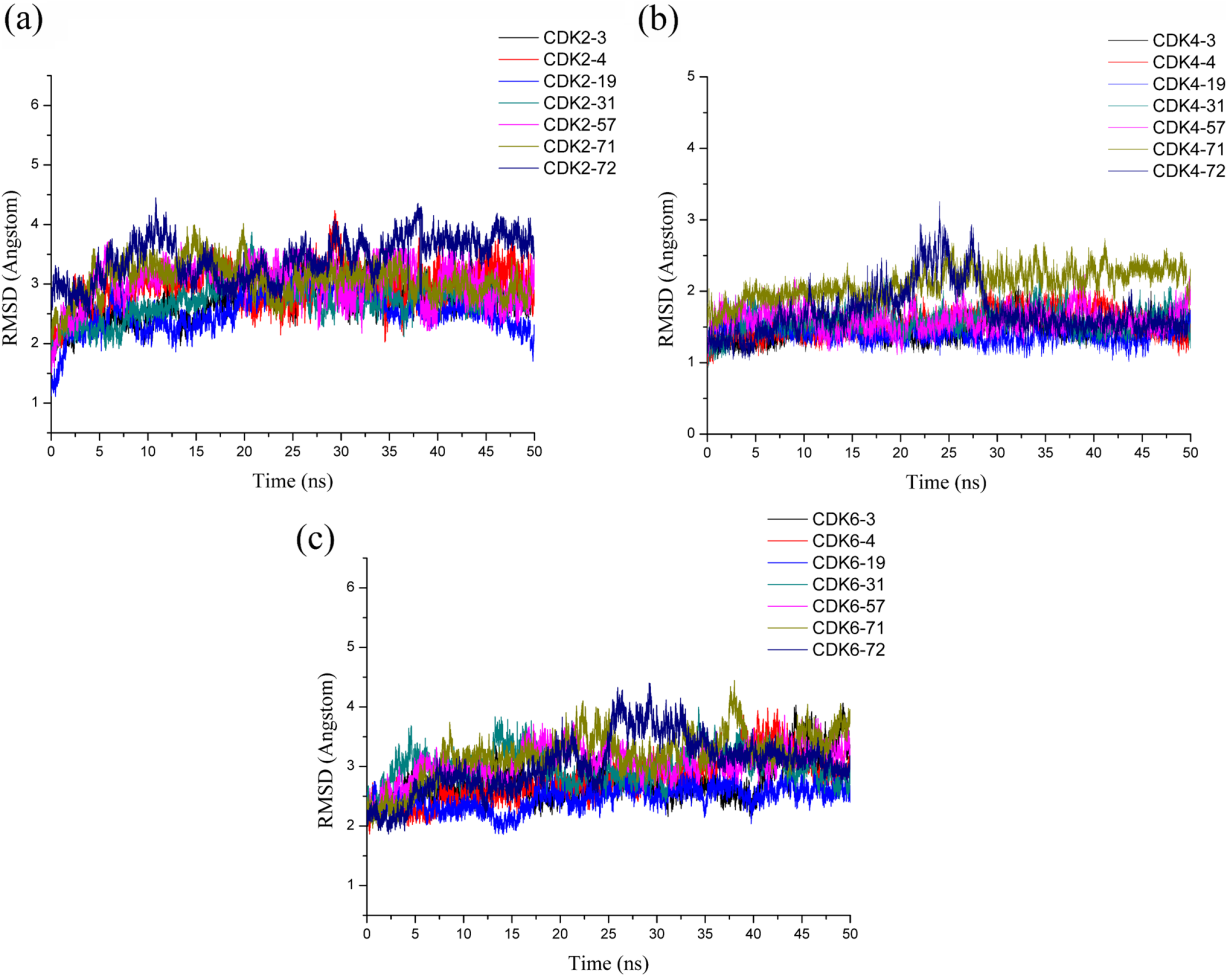
RMSDs of the backbone atoms of the CDK2-inhibitor complexes (**3**, **4**, **19**, **31**, **57**, **71** and **72**) (**a**), CDK4-inhibitor complexes (**3**, **4**, **19**, **31**, **57**, **71** and **72**) (**b**) and the CDK6-inhibitor complexes (**3**, **4**, **19**, **31**, **57**, **71** and **72**) (**c**)

As exhibited in Fig. 7, all systems displayed small RMSD fluctuations, and their average RMSD values were in an acceptable range: 2.55–3.44 Å for the CDK2 systems (see Fig. 7a), 1.42–2.11 Å for the CDK4 systems (see Fig. 7b) and 2.48–3.19 Å for the CDK6 systems (see Fig. 7c). Obviously, the CDK4 system showed greater stability than the CDK2 and CDK6 systems. It was also revealed that the selected compounds tended to have stronger interactions with CDK4 kinase in the ATP-binding pocket than the CDK2 and CDK6 kinases, which might account for the fact that compounds **3**, **4**, **19**, **31**, **57**, **71** and **72** had greater potency against the CDK4 kinase than against the CDK2 and CDK6 kinases.

**Fig. 8:**
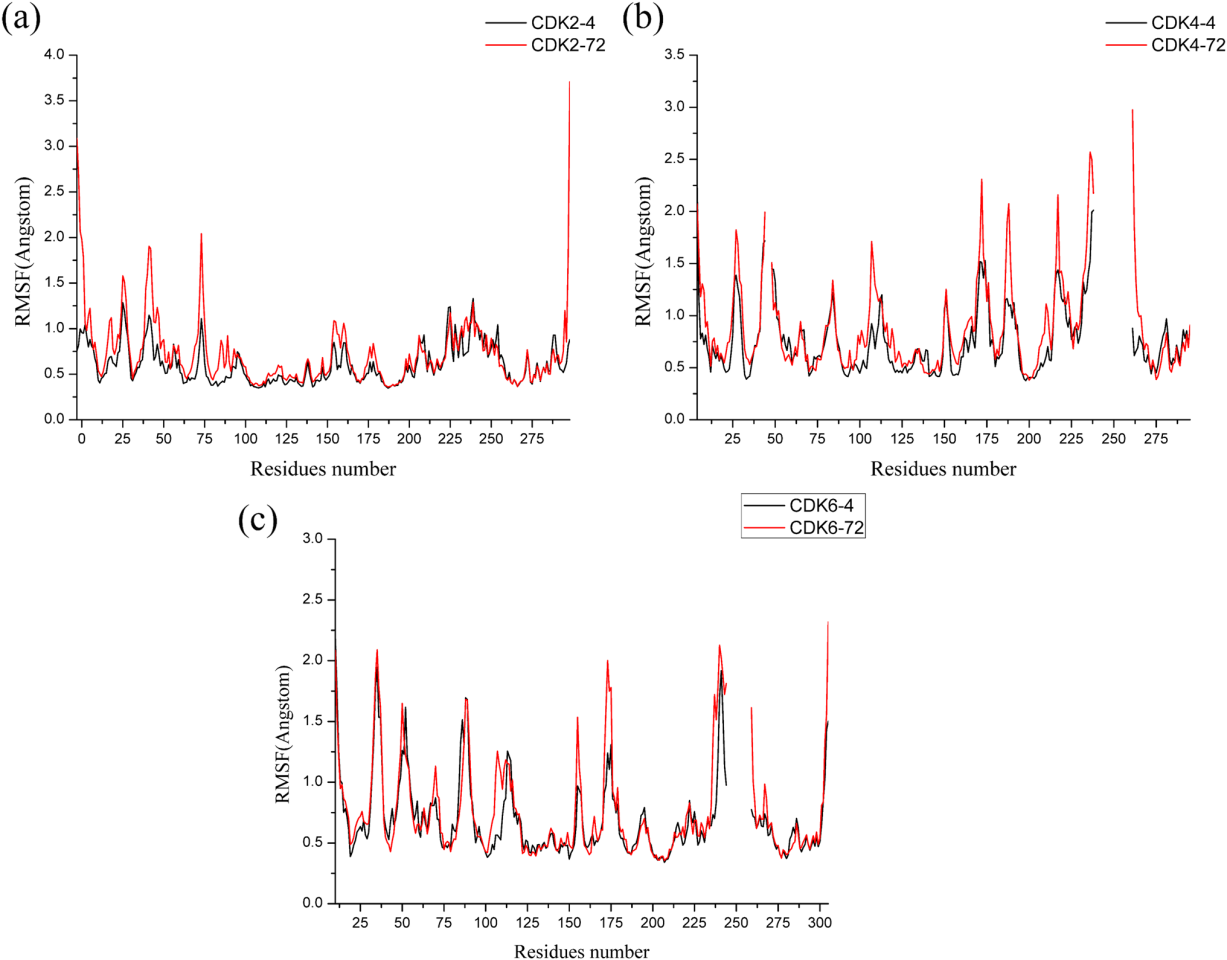
The RMSFs of backbone atoms versus residue numbers of the CDK2-**4** and CDK2-**72** complexes (**a**), CDK4-**4** and CDK4-**72** complexes (**b**) and CDK6-**4** and CDK6-**72** complexes (**c**)

To further identify the interior stability of each residue in the CDK2/4/6 kinases during the modeling dynamics process, the values of root-mean-squared fluctuation (RMSF) of the six systems were computed based on their backbone residues and are displayed in Fig. 6. In the RMSF graphs exhibited in Fig. 8a, we can easily recognize that the backbone residues Ile10, Gly11, Gly13, Leu83, Leu134 and Asp145 in CDK2-**4** and -**72** fluctuated to less than 0.5 Å, which indicated good stability and aligned with the crucial residues identified in our docking result (see Fig. 3a). The other residues showed similar trends, except for Gln85, Asp86 and Lys89, which had much less fluctuation with CDK2-**4** than with CDK2-**72**; for instance, the vibration of Lys89 was up to 0.92 Å for CDK2-**72**, while it was only 0.46 Å for CDK2-**4**. As shown to Fig. 11a, we detected that the positively charged side chain of Lys89 formed strong electrostatic interactions with the protonated nitrogen atom of the R_4_ substituent of compound **4** rather than the aprotic carbon atom of the R_4_ substituent of compound **72**. In Fig. 8b, we found that the backbone residues Ile12, Gly13, Val14 and Val96, Leu146 and Leu147 in CDK4-**4** and CDK4-**72** had satisfying stability due to their strong van der Waals interactions with compounds **4** and **72**. Nevertheless, some differences were observed in both systems, such as for Asp99, Arg101, Thr102 and Asp158, the interactions of which were distinct between the two compounds. For instance, Asp158 served as a donor, interacting with the N_1_H group of the R_4_ substituent in compound **4** instead of compound **72** (see Fig. 11b). For the CDK6-**4** and CDK6-**72** systems (Fig. 8c), the vibrations of some important residues, such as Ile19, Gly20, Val101, Leu152 and Asp163, were small, suggesting that the interactions of these residues were crucial and that both systems showed similar stability. Nonetheless, residues Gln103, Asp104, Thr106 and Thr107, situated at the entrance of the binding site, showed larger fluctuations with CDK6-**72** than CDK6-**4**, specifically at Thr106, the side chain of which formed strong electrostatic interactions with the N_4’’_ atom in compound **4** but not in **72**. Considering these findings, we speculated that the interactions formed by CDK2-**4**, CDK4-**4** and CDK6-**4** became stronger than those formed by CDK2-**72**, CDK4-**72** and CDK6-**72**, which agrees with the experimental bioactivities.

In addition, we further investigated receptor-ligand interaction by monitoring the whole trajectory of the MD simulations for the abovementioned twenty-one systems (Table 4). As shown in Table 4, for three inhibitor pairs (**3**–**57**, **4**–**19** and **31**–**71**), inhibitors **3**, **19**, and **31,** with higher bioactivity, formed more hydrogen bonds with CDK2/4/6 kinases than **57**, **4** and **71**, with lower bioactivity. Furthermore, some hydrogen bonds that formed in the docking model were different than in the MD simulations; for instance, Asn150 C = O…N_1’_H, in the docking model (see Fig. 2e) was replaced by the corresponding hydrogen bond (Asp163 C = O…N_1’_H) in the CDK6-**19** system due to the small movement in the conformation of compound **19**.

**Table 4:** Hydrogen bond analysis from the results of MD simulations

complex	Donor	acceptor	Distance/Å	Angle/(°)	Occupancy/%
CDK2-3	Leu83@O	ligand@N12H	2.193 ± 0.27	32.80 ± 7.59	91.2
	ligand@N9	Leu83@N–H	2.423 ± 0.11	19.41 ± 10.35	98.4
	Asp145@O	ligand@N1’H	3.349 ± 0.21	24.87 ± 8.65	89.6
CDK2-57	Leu83@O	ligand@N12H	2.578 ± 0.18	25.92 ± 9.33	93.6
	ligand@N9	Leu83@N–H	2.651 ± 0.09	36.1 ± 12.51	95.7
CDK4-3	Val96@O	ligand@N12H	2.366 ± 0.13	33.4 ± 14.02	96.8
	ligand@N9	Val96@N–H	2.781 ± 0.19	34.0 ± 16.21	85.8
	Asp158@O	ligand@N1’H	2.537 ± 0.21	27.9 ± 10.54	72.9
CDK4-57	Val96@O	ligand@N12H	2.422 ± 0.14	19.1 ± 8.65	95.5
	ligand@N9	Val96@N–H	2.680 ± 0.23	22.2 ± 10.98	92.1
CDK6-3	Val101@O	ligand@N12H	2.894 ± 0.31	34.7 ± 12.12	93.8
	ligand@N9	Val101@N–H	2.321 ± 0.14	24.81 ± 10.92	88.6
	Asp163@O	ligand@N1’H	3.011 ± 0.23	18.73 ± 9.91	70.1
CDK6-57	Val101@O	ligand@N12H	2.655 ± 0.18	46.84 ± 19.8	96.7
	ligand@N9	Val101@N–H	2.686 ± 0.15	23.90 ± 12.24	90.9
CDK2-4	Leu83@O	ligand@N12H	2.572 ± 0.09	15.80 ± 11.72	95.1
	ligand@N9	Leu83@N–H	2.457 ± 0.13	20.46 ± 9.22	84.8
CDK2-19	Leu83@O	ligand@N12H	2.291 ± 0.16	25.37 ± 10.92	89.2
	ligand@N9	Leu83@N–H	2.502 ± 0.14	38.50 ± 17.11	91.4
	Asp145@O	ligand@N1’H	3.139 ± 0.27	56.41 ± 12.37	68.1
CDK4-4	Val96@O	ligand@N12H	2.470 ± 0.16	47.4 ± 14.32	86.2
	ligand@N9	Val96@N–H	2.887 ± 0.19	51.3 ± 17.94	81.4
CDK4-19	Val96@O	ligand@N12H	2.281 ± 0.11	39.6 ± 15.20	94.7
	ligand@N9	Val96@N–H	2.633 ± 0.14	32.9 ± 14.49	92.3
	Asp158@O	ligand@N1’H	3.045 ± 0.12	23.6 ± 11.32	82.9
CDK6-4	Val101@O	ligand@N12H	2.419 ± 0.15	19.15 ± 7.10	98.9
	ligand@N9	Val101@N–H	2.539 ± 0.21	31.21 ± 10.28	94.1
CDK6-19	Val101@O	ligand@N12H	2.394 ± 0.11	18.33 ± 8.29	99.1
	ligand@N9	Val101@N–H	2.487 ± 0.17	22.97 ± 12.42	95.8
	Asp163@O	ligand@N1’H	2.291 ± 0.12	22.84 ± 11.94	79.3
	ligand@N4	Lys43@N–H	3.025 ± 0.20	47.91 ± 16.85	61.1
CDK2-31	Leu83@O	ligand@N12H	2.574 ± 0.11	26.37 ± 11.92	97.9
	ligand@N9	Leu83@N–H	2.531 ± 0.13	18.66 ± 9.21	98.3
	ligand@N4	Lys33@N–H	3.182 ± 0.25	76.37 ± 18.29	62.5
	Tyr15@O	ligand@N1’H	3.544 ± 0.34	106.7 ± 29.73	42.7
CDK2-71	Leu83@O	ligand@N12H	2.638 ± 0.18	29.81 ± 12.32	96.2
	ligand@N9	Leu83@N–H	2.759 ± 0.12	28.22 ± 14.28	91.8
	Asp145@O	ligand@N1’H	3.289 ± 0.27	36.97 ± 16.11	75.6
CDK4-31	Val96@O	ligand@N12H	2.349 ± 0.13	42.41 ± 18.02	93.2
	ligand@N9	Val96@N–H	2.477 ± 0.16	38.97 ± 14.34	94.8
	Asp158@O	ligand@N1’H	2.172 ± 0.10	26.81 ± 13.02	76.2
CDK4-71	Val96@O	ligand@N12H	2.311 ± 0.15	39.67 ± 11.98	98.1
	ligand@N9	Val96@N–H	3.197 ± 0.28	32.79 ± 16.27	92.9
	Asp158@O	ligand@N1’H	2.783 ± 0.14	38.74 ± 14.88	81.5
CDK6-31	Val101@O	ligand@N12H	2.277 ± 0.17	29.14 ± 10.19	98.8
	ligand@N9	Val101@N–H	2.581 ± 0.20	35.65 ± 12.20	93.4
	Asp163@O	ligand@N1’H	2.749 ± 0.13	34.88 ± 13.72	82.4
	Lys43@N	ligand@N1’H	2.462 ± 0.35	42.49 ± 19.55	31.7
CDK6-71	Val101@O	ligand@N12H	2.364 ± 0.11	26.78 ± 12.86	97.1
	ligand@N9	Val101@N–H	2.609 ± 0.18	33.98 ± 15.31	92.9
	Asp163@O	ligand@N1’H	2.860 ± 0.24	31.15 ± 12.02	70.6
CDK2-72	Leu83@O	ligand@N12H	2.374 ± 0.20	16.81 ± 9.20	97.9
	ligand@N9	Leu83@N–H	2.552 ± 0.13	31.23 ± 14.77	95.6
CDK4-72	Val96@O	ligand@N12H	2.671 ± 0.12	23.95 ± 10.52	98.1
	ligand@N9	Val96@N–H	3.206 ± 0.23	29.11 ± 14.63	96.8
CDK6-72	Val101@O	ligand@N12H	2.591 ± 0.14	32.78 ± 16.72	98.4
	ligand@N9	Val101@N–H	2.988 ± 0.11	25.32 ± 12.84	95.3
	Asp163@O	ligand@N1’H	2.632 ± 0.23	22.37 ± 15.25	72.8
	ligand@N4	Lys43@N–H	3.592 ± 0.38	52.68 ± 24.09	43.2

The binding free energies of the twenty-one systems were determined by the MM-GBSA method, the corresponding statistics of which are displayed in Table 5. Obviously, their binding affinities chiefly aligned with the experimental bioactivities shown in Table 1. To explore the influences of the R_1_ substituents, R_2_ substituents X-positions and R_4_ substituents on the binding affinities in depth four compound pairs (**3**–**57**, **4**–**19**, **4**–**72** and **31**–**71**) and the CDK2/4/6 kinases were methodically compared.

**Table 5:** Binding free energy (kcal/mol) of twenty-one systems

system	Polar contributions			Nopolar contributions			ΔG_bind_ (STD)	K_i_ [μm]
	ΔE_ele_ (STD)	ΔG_ele,sol_ (STD)	ΔE_ele_ + ΔG_ele,sol_ (STD)	ΔE_vdw_ (STD)	ΔG_nonpol,sol_ (STD)	ΔE_vdw_ + ΔG_nonpol,sol_		
CDK2-3	− 23.65 (2.56)	38.37 (2.11) 3	14.72 (1.76)	− 50.05 (2.38)	− 6.43 (0.14)	− 56.48	− 41.76 (2.37)	0.355
CDK2-57	− 20.04 (3.17)	37.50 (2.24)	17.46 (2.15)	− 46.99 (2.29)	− 7.03 (0.13)	− 54.02	− 36.56 (2.63)	1.80
CDK4-3	− 30.24 (3.52)	42.30 (2.75)	12.06 (2.30)	− 57.37 (2.68)	− 7.26 (0.15)	− 64.63	− 52.57 (3.06)	0.002
CDK4-57	− 26.90 (3.93)	40.84 (2.55)	13.94 (2.45)	− 54.28 (2.58)	− 7.10 (0.13)	− 61.38	− 47.44 (2.52)	0.010
CDK6-3	− 24.02 (4.28)	39.95 (3.54)	15.93 (3.21)	− 53.84 (2.78)	− 7.61 (0.16)	− 61.45	− 45.52 (3.38)	0.011
CDK6-57	− 15.52 (3.52)	36.07 (3.23)	20.55 (2.54)	− 50.58 (3.50)	− 6.59 (0.19)	− 57.17	− 36.62 (4.15)	1.67
CDK2-72	− 17.67 (2.87)	38.21 (2.29)	20.54 (1.97)	− 50.94 (2.43)	− 7.01 (0.14)	− 57.95	− 37.41 (2.45)	5.00
CDK2-4	− 20.42 (2.56)	37.77 (2.11)	17.35 (1.86)	− 51.73 (2.38)	− 7.11 (0.24)	− 58.84	− 41.49 (2.37)	0.776
CDK2-19	− 27.48 (4.16)	42.21 (3.53)	14.73 (2.43)	− 53.35 (2.72)	− 7.07 (0.15)	− 60.42	− 45.69 (3.05)	0.014
CDK4-72	− 20.62 (2.87)	42.18 (2.29)	21.56 (2.52)	− 56.17 (2.43)	− 7.97 (0.14)	− 64.14	− 42.58 (2.45)	0.070
CDK4-4	− 28.55 (3.52)	45.47 (3.65)	16.92 (2.73)	− 57.52 (2.78)	− 7.59 (0.16)	− 65.11	− 48.19 (3.52)	0.006
CDK4-19	− 36.10 (4.32)	48.91 (3.00)	12.81 (2.49)	− 57.79 (2.26)	− 7.56 (0.19)	− 65.35	− 52.54 (3.17)	0.004
CDK6-72	− 18.60 (3.09)	37.52 (2.93)	18.92 (2.50)	− 51.43 (2.82)	− 6.87 (0.18)	− 58.30	− 39.38 (3.09)	0.257
CDK6-4	− 20.61 (4.40)	37.21 (2.74)	16.60 (3.16)	− 51.71 (3.21)	− 7.35 (0.21)	− 59.06	− 42.46 (4.95)	0.093
CDK6-19	− 28.17 (4.16)	41.12 (2.77)	12.95 (3.05)	− 52.71 (3.75)	− 7.74 (0.15)	− 60.45	− 47.50 (3.46)	0.006
CDK2-31	− 22.86 (3.78)	39.73 (2.95)	16.87 (1.96)	− 52.12 (2.93)	− 7.13 (0.32)	− 59.25	− 42.38 (3.40)	0.037
CDK2-71	− 19.98 (2.88)	38.16 (3.63)	18.18 (2.52)	− 48.88 (2.71)	− 6.85 (0.14)	− 55.73	− 37.55 (2.61)	5.00
CDK4-31	− 22.31 (3.39)	39.58 (2.54)	17.27 (2.44)	− 55.75 (2.52)	− 7.41 (0.17)	− 63.16	− 45.89 (2.63)	0.006
CDK4-71	− 24.28 (3.53)	40.50 (3.17)	16.22 (2.47)	− 52.46 (2.90)	− 6.67 (0.20)	− 59.13	− 42.91 (3.90)	0.169
CDK6-31	− 20.83 (3.78)	37.19 (3.11)	16.36 (2.46)	− 51.39 (3.11)	− 6.74 (0.10)	− 58.13	− 41.77 (3.05)	0.225
CDK6-71	− 20.12 (3.09)	37.82 (3.42)	17.61 (2.31)	− 49.63 (2.55)	− 5.47 (0.09)	− 55.10	− 37.49 (2.91)	2.710

All energies are in kcal/mol

Comparing compounds **3** and **57**, only their R_1_ substituents were different. Compound **3** featured a -N_1’_H-cyclopentyl group at the R_1_ substituent, whereas in compound 57, it was replaced by a -CH_3_ group, indicating that the former showed better potency than the latter with CDK2/4/6 kinases. This outcome was proven by the results of the binding free energy, as the ΔG_bind_ values of CDK2-**3** (− 41.76 kcal/mol), CDK4-**3** (− 52.57 kcal/mol) and CDK6-**3** (− 45.52 kcal/mol) were dramatically higher than those of CDK2-**57** (− 36.56 kcal/mol), CDK4-**57** (− 47.44 kcal/mol), and CDK6-**57** (− 36.62 kcal/mol), which indicated that compound **3** interactions with CDK2/4/6 kinases were more stable than those of compound **57**. In Fig. 8a–c, compound **3** formed three hydrogen bonds with Asp145 of CDK2, Asp158 of CDK4 and Asp163 of CDK6. However, these hydrogen bonds in CDK2-57, CDK4-57 and CDK6-57 were nonexistent, which coincided with the CoMFA results showing the introduction of an electronegative atom at the N_1’_-position was favorable for inhibitor bioactivity. This finding was also supported by the contributions of polarity (ΔG_ele_ + ΔG_ele,sol_), as shown in Table 5, where the polar contributions (ΔG_ele_ + ΔG_ele,sol_) of CDK2-**3** (14.72 kcal/mol), CDK4-**3** (12.06 kcal/mol) and CDK6-**3** (15.93 kcal/mol) were distinctly stronger than those of CDK2-**57** (17.46 kcal/mol), CDK4-**3** (13.94 kcal/mol) and CDK6-**3** (20.55 kcal/mol). As shown in Fig. 8d and g, the CDK2 systems demonstrated that the key residues Gly11, Glu12, Gly13, Lys33, Lys89 and Asp145 clearly contributed more to **3** than to **57**, particularly Asp145, whose difference of energy in compounds **3** and **57** reached − 1.53 kcal/mol. Moreover, the positively charged side chain of Lys89 formed a stronger electrostatic interaction with compound **3** than with **57** because of the short distance from and spatial orientation with the N_4’’_ atom. For the CDK4 system (Fig. 8b, e and h), the crucial residues accounting for the energy differences between compounds **3** and **57** were Gly13, Val14, Gly15, Arg101 and Asp158, and their energy differences were essentially derived from their nonpolar interactions (ΔG_vdw_ + ΔG_nonpol,sol_). Here, Val14, contacting R_1_ directly_,_ formed distinctly stronger hydrophobic interactions with compound **3** than with compound **57**, further proving that the nonpolar interactions were the major contributors. The nonpolar interaction of **3** (− 64.63 kcal/mol) was notably higher than that of **57** (− 61.38 kcal/mol) in Table 5. Thus, we regarded the nonpolar interaction as an important factor affecting the different bioactivities of compound **3** and **57** against CDK4. With regard to the two CDK6 systems (Fig. 8c, f and I), we also discovered that the favorable contributions of Gly20, Glu21, Gly22, Asp104, Thr106, Thr107 and Asp163 for compound **3** were much larger than those for compound **57**, but Lys43, positioned in closer vicinity to the N_4_ atom of compound **57,** formed a stronger polar interaction with **57** than with **3**. Nonetheless, the increased polar interactions of compound **57** with Lys43 were not sufficient to make up for the weakening interactions with other residues. This finding was in accordance with their binding free energy, as shown in Table 5, where the ΔG_bind_ of CDK6-**3** was − 8.90 kcal/mol over CDK6-**57**. As seen in Fig. 8c, the nonpolar residues Gly20 and Gly22 were located close to the R_1_ of **3**, where the cyclopentyl ring easily contacted Gly20 and Gly22 through strong nonpolar interactions. In addition, the polar residues Asp104, Thr106 and Thr107 were situated around the protonated N_4’’_ atom and established stronger polar interactions with **3** than **57** due to their closer distance and favorable orientation to ring D. In summary, the polar and nonpolar interactions jointly influenced the bioactivity of compounds **3** and **57** against CDK2/4/6 kinases. We also concluded that possessing an electron at the N_1’_ position and replacing ring E with a moderately sized hydrophobic group was important for designing the R_1_ substituent.

**Fig. 9:**
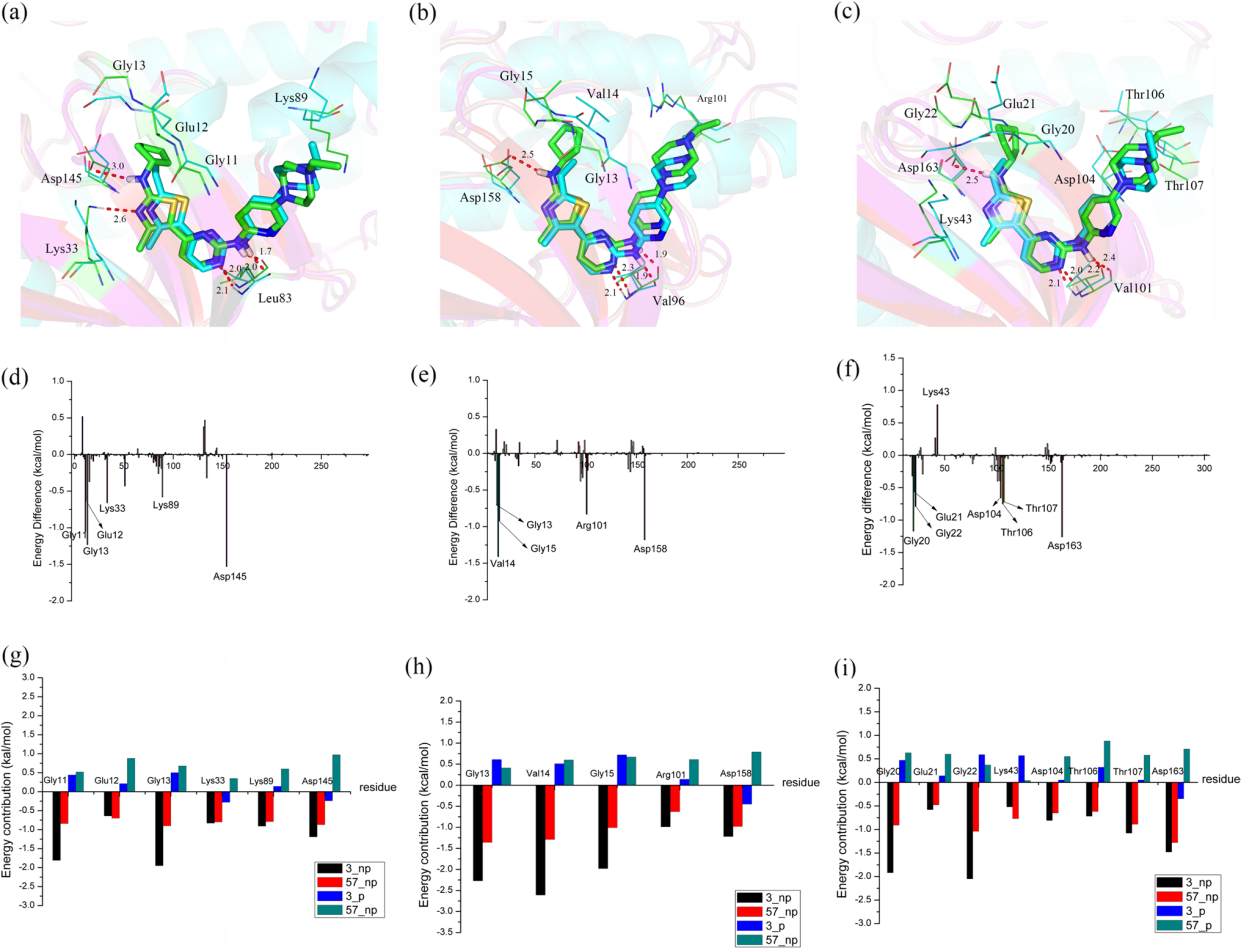
Comparison of the basic structures of the CDK2-**3** and CDK2-**57** (**a**), CDK4-**3** and CDK4-**57** (**b**) and CDK6-**3** and CDK6-**57** (**c**) complexes (carbon atoms are shown in green for **3** and in cyan for **57**); energy differences of each residue in the binding of CDK2-**3** and CDK2-**57** (**d**), CDK4-**3** and CDK4-**57** (**e**) and CDK6-**3** and CDK6-**57** (**f**); the contributions of the individual energy terms for the key residues (**g**, **h** and **i**, the black and red columns represent ΔGvdw and ΔGnonpol,sol, respectively, and the blue and cyan columns represent ΔGele and ΔGele,sol, respectively)

The chemical structures of compounds **4** and **19** were similar except for the R_2_ substituent, where –CF_3_ in **19** was substituted with –CH_3_ in **4**. Obviously, compound **19** likely has stronger polar-interactions than **4** with CDK2/4/6 kinases. As displayed in Table 5, the polar energy was found to make the prominent contribution to the binding with three receptors, where the polar tendency (ΔG_ele_ + ΔG_ele,sol_) of CDK2-**19** was − 4.20 kcal/mol, which was greater than that of CDK2-**4**, and that of CDK4-**19** was − 4.35 kcal/mol greater than that of CDK-**4** and that of CDK4-**19** was − 5.04 kcal/mol greater than that of CDK-**4**. For the CDK2-**19** and CDK2-**4** systems, Fig. 9d and g shows that five important residues, Tyr19, Lys33, Glu51, Lys89 and Asp145, showed large energy differences, and these residues are polar residues, especially Lys33, which was within the proximity of –CF_3_, whose energy difference between **19** and **4** reached − 1.52 kcal/mol. As shown in Fig. 9a and g, in contrast with compound **4**, compound **19** formed a hydrogen bond with Asp145, resulting in Asp145 linking compound **19** with distinctly higher polar interaction than compound **4**. For two CDK4 systems (Fig. 9b, e and h), the polar residues Tyr21, Lys35, Asp99, Arg101 and Asp158 made greater contributions for binding **19** than for **4**, but Thr102 was located in closer proximity to the C = O of **4** and formed a stronger polar interaction with **4** than with **19**. However, **19** still had stronger polar interactions than **4** with CDK4 kinase. As illustrated in Fig. 9b, we observed that compound **19** formed four hydrogen bonds with Lys35, Leu96 and Asp158, while compound **4** formed only three hydrogen bonds with Leu96 and Asp158. Furthermore, the nonpolar interaction of **19** (− 65.35 kcal/mol) had a similar contribution as 4 (− 65.11 kcal/mol), as shown in Table 5, which further indicated that the polar interaction primarily accounted for the difference in the bioactivities of compounds **19** and **4** against CDK4 kinase. With respect to the CDK6-**19** and CDK6-**4** systems, as shown in Fig. 9f and I, the significant residues Lys43, Glu61, Thr106 and Asp163 made greater beneficial contributions toward the different bioactivities between **19** and **4**. Lys43 and Asp163, displayed the largest energy difference of **19** over that of **4**, formed additional hydrogen bonds with **19**. In addition, Lys43 was positioned close to R_2_ and contacted the –CF_3_ group of **19** through stable electrostatic interactions, but not the –CH_3_ group of 4. Moreover, the nonpolar interaction was roughly equal in the two systems (− 60.45 kcal/mol for **19** and − 59.06 kcal/mol for **4**), as shown in Table 5, implying that the polar interaction was responsible for the energy difference of CDK6-**19** and CDK6-**4**. These data indicated that retaining the –CF_3_ group as an R_2_ substituent was an effective approach for increasing the inhibitor bioactivity against CDK2/4/6 kinases.

**Fig. 10:**
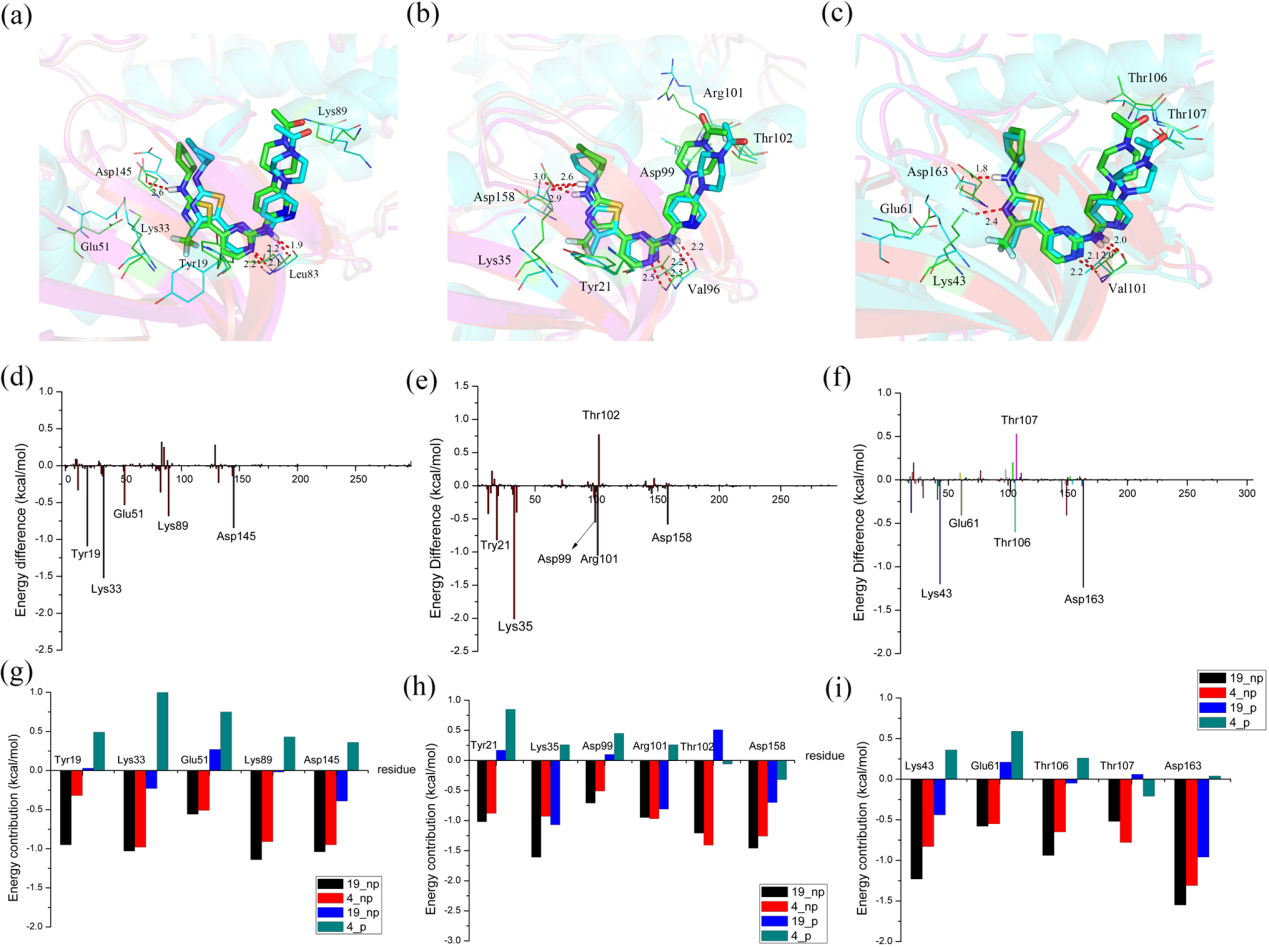
Comparison of the averaged structures of the CDK2-**4** and CDK2-**19** (**a**), CDK4-**4** and CDK4-**19** (**b**) and CDK6-**4** and CDK6-**19** (**c**) complexes (carbon atoms are shown in green for **4** and in cyan for **19**); energy difference of each residue in the binding of CDK2-**4** and CDK2-**19** (**d**), CDK4-**4** and CDK4-**19** (**e**) and CDK6-**4** and CDK6-**19** (**f**); the contributions of the individual energy terms for the key residues (**g**, **h** and **i**, the black and red columns represent ΔGvdw and ΔGnonpol,sol, respectively, and the blue and cyan columns represent ΔGele and ΔGele,sol, respectively)

The R_3_ substituent in compounds **4** and **72** was different; it was an –NCOCH_3_ group in **4** and a –CH_2_ group in **72**. The much greater bioactivity of **4** compared to that of **72** reveals that the –NCOCH_3_ group, with strong hydrophilic properties, showed favorable binding to CDK2/4/6 kinases. Accordingly, we also speculated that the electrostatic interactions between the –NCOCH_3_ group and surrounding residues might be stronger. This supposition was proven by the results from the binding energy calculations (Table 5), showing that the polar interactions of CDK2-**4** (17.35 kcal/mol), CDK4-**4** (16.92 kcal/mol) and CDK6-**4** (16.60 kcal/mol) were markedly higher than those of CDK2-**72** (20.54 kcal/mol), CDK4-**72** (21.56 kcal/mol) and CDK6-**72** (18.92 kcal/mol). As displayed in Fig. 10a, d and g, for two CDK2 systems, the residues critical for the energy difference were Gln85, Asp86 and Lys89, which all surrounded the R_3_ substituent and are polar residues, particularly Lys89, whose –NH group, with very close proximity to the protonated N_4’’_ atom of the –NCOCH_3_ group, established stable electrostatic interactions with compound **4**. Similar to CDK2 systems, the residues around R_3_ (Gln98, Asp99, Arg101 and Thr102) also primarily contributed to the different energies of the CDK4 systems (see Fig. 10b, e and h). This was especially true for Thr102, regarded as the residue with the largest energy difference between **4** and **72**; its –NH group was able to form a strong electrostatic interaction with the protonated N_4’’_ atom of the –NCOCH_3_ group because of the small distance and dimensional orientation to the –NCOCH_3_ group. In addition, Asp158 contributed obvious differences in energy up to − 0.77 kcal/mol due to its greater ability to form hydrogen bonds with the N_1’_ atom of compound **4** than with **72**. In contrast, it is shown in Fig. 10c that the C = O moiety of Asp163 formed an additional hydrogen bond with the N_1’_ atom of compound **72** instead of **4** in CDK6 systems. This bond caused the polar interaction of Asp163 with compound **72** to be much higher than it was with **4** (see Fig. 10i). Moreover, we also observed that Lys43 was adjacent to compound **72** and established stronger polar interactions with **72** than with **4**. Nevertheless, the increasing number of polar interactions of compound **72** with Lys43 and Asp163 were not sufficient to compensate for the impaired interactions with the residues surrounding the R_3_ substituent (Gln103, Asp104, Thr106 and Thr107), which were all polar residues and made distinctly more favorable contributions to **4** than to **72**. For instance, Thr107, whose C=O moiety was orientated toward the protonated N_4’’_ atom of compound **4**, was clearly more tightly connected with **4** than with **72** because of the strong polar interaction. Through the above results, it was confirmed that the difference in polar interactions led to the higher bioactivity of **4** over **72** against the CDK2/4/6 kinases. Thus, we should preserve the protonated nitrogen atom at the N_4’’_ position and pay more attention to the interactions with Lys89 of CDK2, Arg101 of CDK4 and Thr107 of CDK6 when designing R_3_ substituents.

**Fig. 11:**
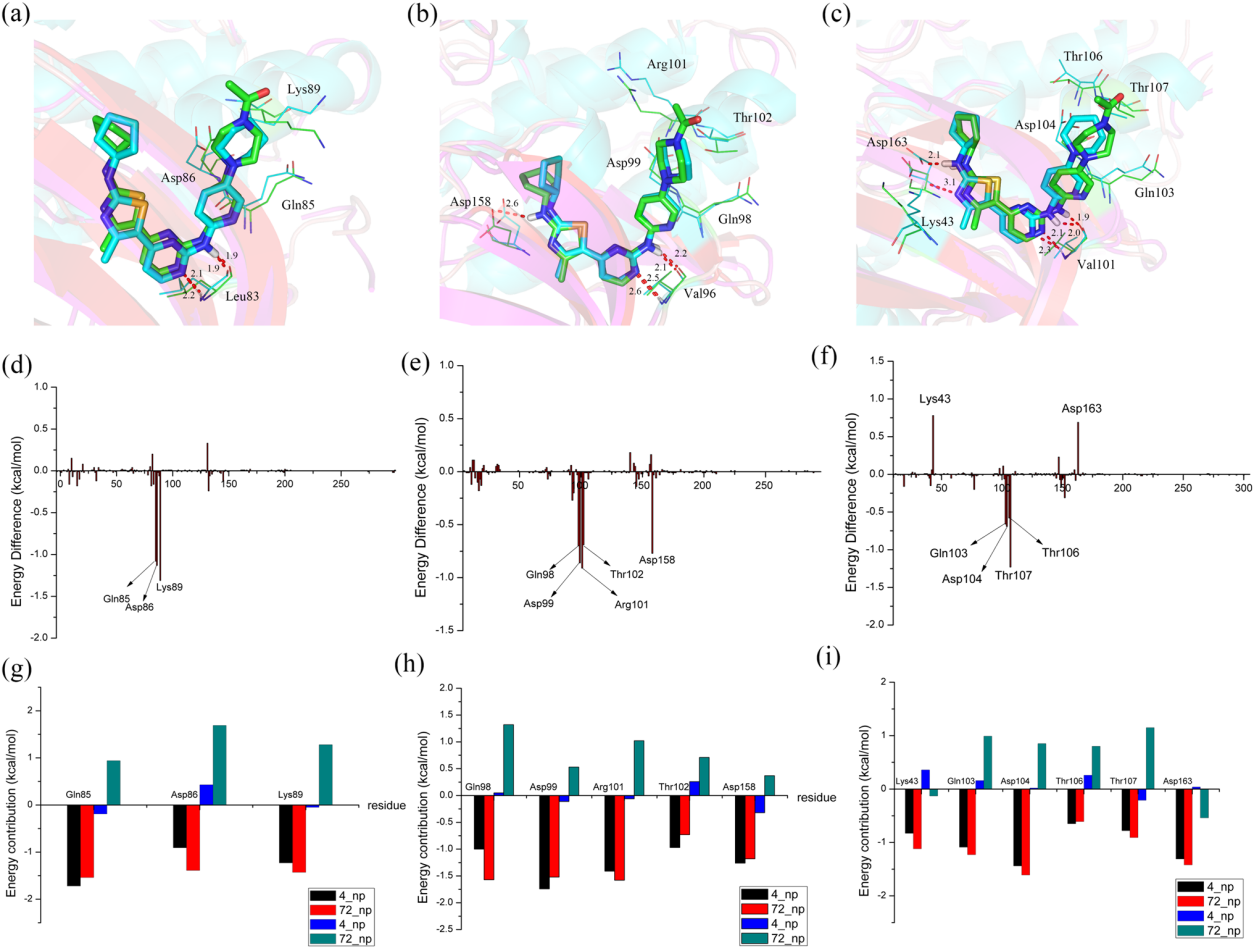
Comparison of the averaged structures for the CDK2-**4** and CDK2-**72** (**a**), CDK4-**4** and CDK4-**72** (**b**) and CDK6-**4** and CDK6-**72** (**c**) complexes (carbon atoms are shown in green for **4** and cyan for **72**, respectively); energy difference of each residue to the binding of CDK2-**4** and CDK2-**72** (**d**), CDK4-**4** and CDK4-**72** (**e**) and CDK6-**4** and CDK6-**72** (**f**); the contributions of the individual energy terms for the key residues (**g**, **h** and **i**, the black and red columns represent ΔGvdw and ΔGnonpol,sol, respectively, and the blue and cyan columns represent ΔGele and ΔGele,sol, respectively)

**Fig. 12:**
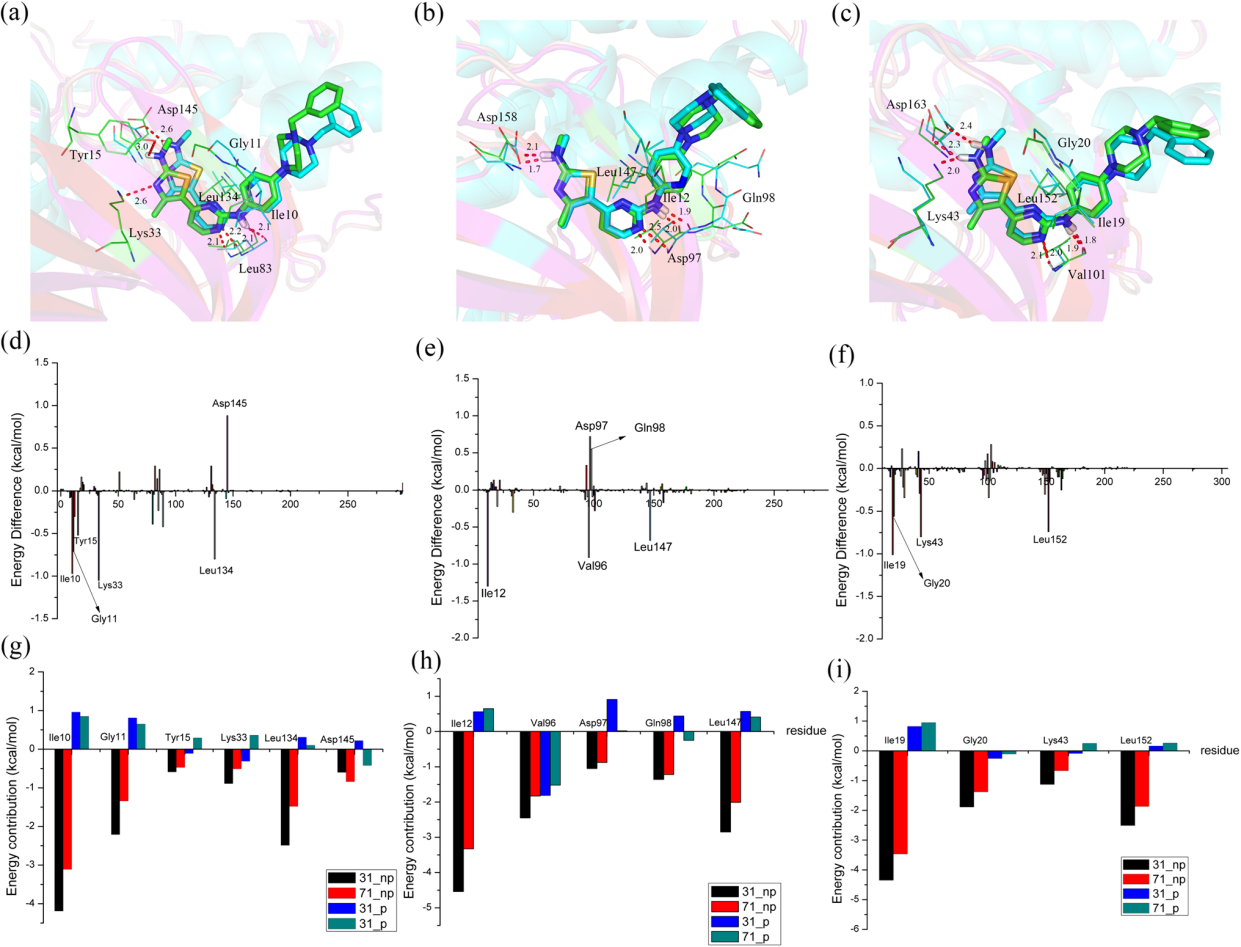
Comparison of the averaged structures for the CDK2-**31** and CDK2-**71** (**a**), CDK4-**31** and CDK4-**71** (**b**) and CDK6-**31** and CDK6-**71** (**c**) complexes (carbon atoms are shown in green for **31** and cyan for **71**, respectively); energy difference of each residue to the binding of CDK2-**31** and CDK2-**71** (**d**), CDK4-**31** and CDK4-**71** (**e**) and CDK6-**31** and CDK6-**71** (**f**); the contributions of the individual energy terms for the key residues (**g**, **h** and **i**, the black and red columns represent ΔGvdw and ΔGnonpol,sol, respectively, and the blue and cyan columns represent ΔGele and ΔGele,sol, respectively)

The difference between compounds **31** and **71** was at the X-position of ring **C**, where a carbon atom in **31** was displaced by a nitrogen atom in **71**. Accordingly, the ring **C** moieties of **31** and **71** were benzene and pyridine rings, respectively. For the benzene ring, its hydrophobic properties were comparable with those of a pyridine ring, near which the hydrophobic residues tended to form powerful nonpolar interactions. Then, as shown in Fig. 11a–c, we found that Ile10, Gly11 and Leu134 of CDK2, Ile12 and Leu147 of CDK4, and Ile19, Gly20 and Leu152 of CDK6 were located close to ring C. These residues all showed high hydrophobic properties and formed strong nonpolar interactions with benzene rings instead of pyridine rings. Therefore, Fig. 11d–i clearly demonstrates that the nonpolar residues Gly11 and Leu134 of CDK2, Ile12 and Leu147 of CDK4, and Ile19, Gly20 and Leu152 of CDK6 all contributed more to **31** than to **71**. As illustrated in Table 5, the nonpolar interactions were also recognized as the major contributors of energy differences between **31** and **71**. Polar interactions also partly influenced energy differences. For two CDK2 systems (Fig. 11a, d and g), Tyr15 and Lys33 contributed more polar interactions to **31** than to **71**, while Asp145 was connected more tightly with **71** than with **31** through polar interactions. Except at Val83, **31** can form two hydrogen bonds with Tyr15 and Lys33, **71** forming one hydrogen bond with Asp145. As displayed in Table 5, the polar interactions of **31** were slightly higher than those of **71**. With regard to the CDK4-**31** and CDK-**71** systems (Fig. 11b, e and i), the polar residues Asp97 and Gln98 lay adjacent to the N_14_ atom of the pyridine ring and formed substantially stronger polar interactions with **71** than with **31**, which might partly explain why the bioactivity of **31** against CDK**4** was nearly **tenfold** higher than that of **71** against CDK2 and CDK6. For CDK6 systems (Fig. 11c, f, and i), an additional strong hydrogen bond between **31** and Lys43 caused a greater number of polar interaction for **31** than for **71**, which further enhanced the stability of CDK4-**31** in comparison with that of CDK4-**71**. Based on the above analysis, we concluded that both nonpolar and polar interactions, especially nonpolar interactions, played important roles in causing the different bioactivities of compounds **31** and **71** against CDK2/4/6 kinases. Therefore, similar to compound **31**, the whole series of compounds should possess a carbon atom at the X- position or the pyridine ring should be replaced with a benzene ring, changes that were very promising for increasing bioactivity (Fig. 12).

## Discussion

In this present study, we analyzed the interactions and structure–activity relationships of 72 novel CDK2/4/6 inhibitors with the comprehensive approaches of molecular docking, 3D-QSAR and MD simulations. Molecular docking results demonstrated that the studied inhibitors contacted three receptors with similar modes, with some small differences. For instance, the cyclopentyl groups of inhibitors formed strong hydrophobic interactions with Val14 of CDK4 but not Glu12/Glu21 of CDK2/6. The reliability of docking modes for CDK2/4/6 and the significant substituent features (R_1_, R_2_, R_4_ and X) were further verified by MD simulations. The constructed 3D-QSAR models of the three receptors were reasonable and confirmed by the excellent statistical data (q^2^ = 0.714 and *R*^2^_pred_ = 0.764 for CDK2; q^2^ = 0.815 and *R*^2^_pred_ = 0.681 for CDK4; and q^2^ = 0. 757 and *R*^2^_pred_ = 0.674 for CDK6). These models had dependable internal verifiability and external predictability. MD simulation and decomposition energy analysis further recognized the interesting nonpolar and polar interactions of CDK2/4/6 receptors by comparing the interactions between four selected pairs of inhibitors (3–57, 4–19, 4–72, 31–71) and the three receptors. According to the integrated results of the docking, 3D-QSAR and MD simulations, we ultimately concluded some groups featured in the corresponding locations probably enhanced the bioactivity against CDK2/4/6 kinases.

## Conclusion

The constructed 3D-QSAR models of the three receptors were reasonable and confirmed by the excellent statistical data. According to the integrated results of the docking, 3D-QSAR and MD simulations, we ultimately concluded that the following groups featured in the corresponding locations probably enhanced the bioactivity against CDK2/4/6 kinases: (1) electronegative groups at the N1-position and electropositive and moderate-sized groups at ring E; (2) electrogroups featured at R2; (3) carbon atoms at the X-position or ring C replaced by a benzene ring; and (4) an electrogroup as R4. Finally, we hope the results obtained from this work will provide some useful references for the development of novel CDK2/4/6 inhibitors.

## Data Availability

The datasets generated during and analysed during the current study are available in the RSCB Protein Data Bank repository, https://www.rcsb.org/.
